# A Comparative Study of Forward Osmosis Systems: An Integrated Experimental, Modeling, and Techno-Economic Analysis

**DOI:** 10.1021/acsomega.6c07030

**Published:** 2026-09-15

**Authors:** Aicha Gasmi, Sabrine Chamam, Soumaya Ibrahimi, Milène Chroudi, Marc Heran, Ahmed Hannachi

**Affiliations:** † Laboratory of Engineering Processes and Industrial Systems, Chemical Engineering Department, National School of Engineering of Gabes, 198826 University of Gabes, 6029 Gabes, Tunisia; ‡ 27037 Institut Européen des Membranes—IEM, UMR 5635, Univ Montpellier, CNRS, ENSCM, Montpellier 34095, France

## Abstract

Draw solution chemistry is a design parameter in forward osmosis (FO), governing hydraulic performance, organic rejection, fouling dynamics, and process economics, yet these dimensions are rarely evaluated together, particularly under wastewater feed conditions. This study addresses that gap through an integrated experimental, modeling, and techno-economic framework using three draw solutions (NaCl, MgSO_4_, and sodium acetate), each prepared at an initial osmotic pressure of 30 bar, and two feed solutions (deionized water (DI) and real municipal wastewater (WW)). Water flux (*J*_w_), reverse salt flux (*J*_s_), and chemical oxygen demand (COD) flux (*J*_COD_) were monitored over 4 hours. Water fluxes ranged from 1.8 to 4.0 L m^–2^ h^–1^, and substituting DI with WW reduced *J*_w_ by 8.1–46.2% depending on draw solution chemistry. A dynamic MATLAB finite-difference model coupling concentration polarization with membrane transport accurately reproduced experimental *J*_w_ and *J*_s_ profiles, with Root Relative Mean Square Error below 1.9% and 6%, respectively. A phenomenological COD sub-model partitioned organic matter dynamics into three pathways and introduced a dimensionless ratio *P*_COD_/*K*_foul_ as a selectivity screening metric. Techno-economic analysis identified NaCl as the most economically viable draw solution, achieving a levelized cost of water of USD 1.04 m^–3^. Monte Carlo analysis confirmed this advantage was statistically robust with complete non-overlap of NaCl ’s 95% confidence interval and those of MgSO_4_ and sodium acetate. MgSO_4_ demonstrated superior COD rejection, lower initial fouling accumulation, and more stable flux decay kinetics within the initial-to-intermediate fouling regime examined, making it preferable where selectivity and effluent quality are prioritized.

## Graphical abstract

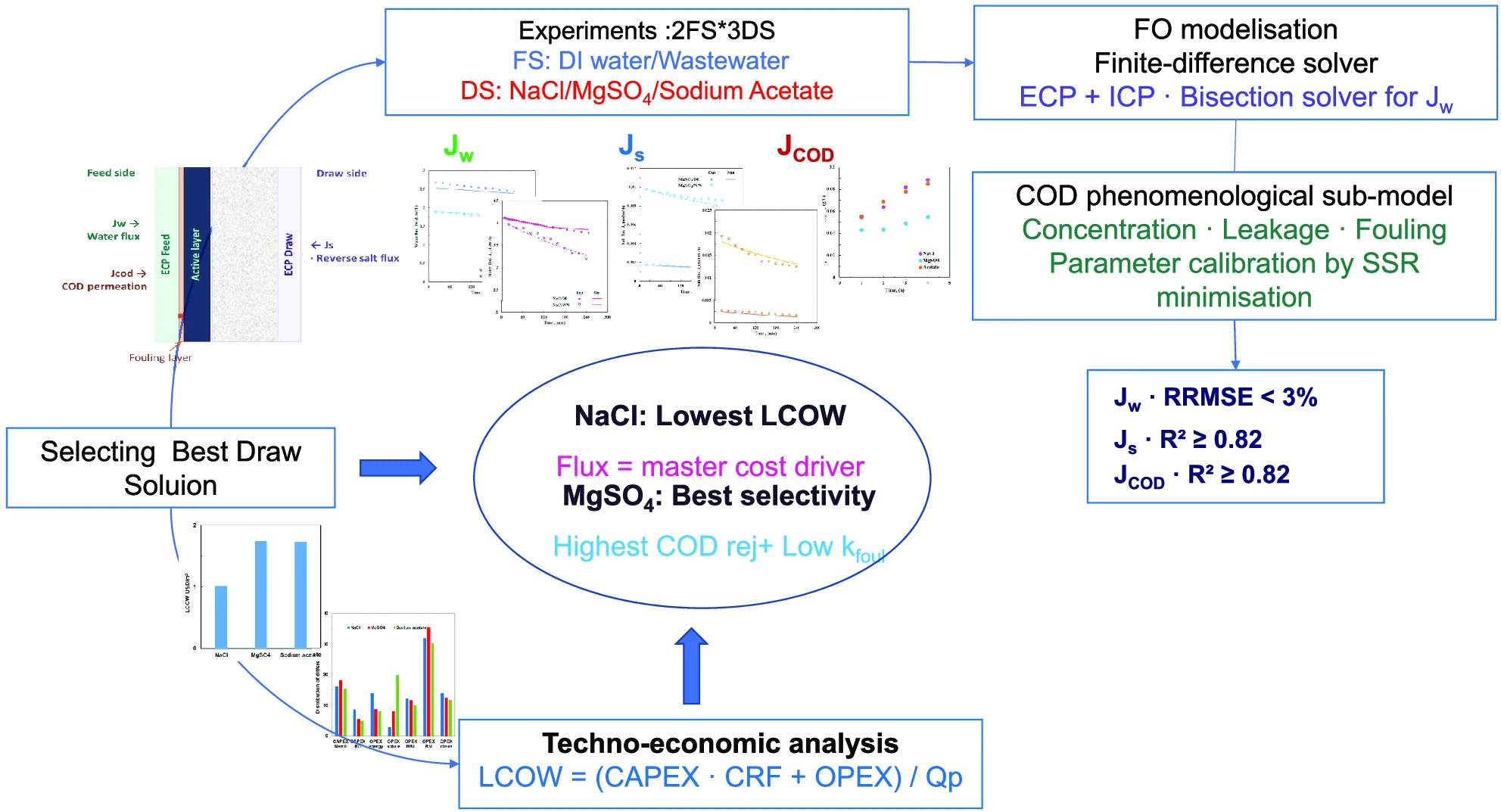

## 1 Introduction

Forward Osmosis (FO), driven by the osmotic pressure difference rather than applied hydraulic pressure, has attracted sustained interest for wastewater treatment owing to its lower membrane compaction, reduced specific energy consumption, and potential for integration with renewable energy sources.^1–3^ The selection of the draw solution (DS) is the central design variable in any FO system. It simultaneously governs the osmotic driving force, the reverse solute flux that degrades feed quality, the concentration polarization (CP) regime, and the cost of downstream regeneration. Despite this centrality, no study has yet compared draw solution chemistries across hydraulic performance, organic rejection, fouling, and economics under real wastewater feed conditions within a single, unified predictive framework.^4–6^

Three coupled phenomena constrain FO performance and complicate DS comparison: Reverse solute diffusion, CP, and fouling.^4–6^ Reverse solute diffusion, the leakage of DS components into the feed solution (FS), reduces the osmotic driving force and aggravates fouling.^7,8^ Currently, it is best mitigated by selecting larger, slow-diffusing, or thermo-responsive solutes.^4^ Independently, CP further attenuates the effective osmotic driving force and is more severe in FO than in pressure-driven processes.^9,10^ CP manifests as concentrative external CP (ECP) at the active layer and, in the active-layer-facing-feed orientation adopted here, dilutive internal CP (ICP) within the support layer, the latter being less amenable to hydrodynamic mitigation.^11^ Although FO experiences less membrane fouling than reverse osmosis (RO) because of lower operating pressure,^12,13^ complex real feeds still induce time-varying organic deposition, biofouling, and colloidal cake formation that progressively reduce flux and compromise membrane integrity, introducing organic permeation pathways that steady-state models cannot capture.^5,7^ RSD, CP, and fouling do not act independently: RSD amplifies CP, which in turn promotes fouling and further attenuates the driving force. Because conventional experimental designs average these contributions into a single response, the individual contribution of each mechanism and its dependence on draw solution chemistry and feed matrix complexity remain poorly resolved. Thus, evaluating draw solutions against any single metric risks misrepresenting their comparative performance under realistic conditions.

Prior techno-economic assessments of FO have generally been developed independently of draw-solution-specific transport behavior. Pure transport comparisons across multiple draw solutions, such as those by Achilli et al.^14^ and Ge et al.,^4^ provide no economic dimension, while existing economic analyses either focus on a single draw solution or adopt literature-based flux values rather than experimentally calibrated transport parameters.^15–21^ No prior study has simultaneously applied a parameter-level calibration of levelized cost of water against experimentally resolved *J*_w_, *J*_s_, and *K*_foul_ across three chemically distinct draw solutions under real municipal wastewater feed conditions. Accordingly, integrating experimental observations with mechanistic modeling and parameter-calibrated economic evaluation is essential for a quantitative and actionable draw solution selection framework under realistic operating conditions.

This study addresses these gaps through an integrated three-pillar framework applied to three draw solutions (NaCl, MgSO_4_, and sodium acetate) prepared at identical initial osmotic pressure. Each DS was tested against two FS (deionized water and wastewater) to isolate the flux reduction and fouling imposed by a complex feed matrix and to quantify their effects on water flux (*J*_w_), reverse salt flux (*J*_s_), and chemical oxygen demand (COD) flux (*J*_COD_). Simultaneously, a dynamic MATLAB model, coupling ICP and ECP with membrane transport equations was developed to predict the time-resolved FO performance, thereby enabling mechanistic discrimination between osmotic driving force losses and membrane-selectivity limitations. A phenomenological COD sub-model was further integrated into this framework to quantify organic matter partitioning among three pathways: feed-side COD concentration enhancement arising from volume reduction by transmembrane water extraction, transmembrane leakage, and irreversible fouling deposition. A dimensionless ratio *P*_COD_/*K*_foul_ was introduced as a single-parameter selectivity screening metric for draw solution comparison, quantifying the relative dominance of organic leakage over foulant deposition. Building on these transport results, the experimentally measured fluxes and fouling parameters were used to calibrate a techno-economic framework, from which levelized cost of water (LCOW) probability distributions were derived via Monte Carlo uncertainty propagation. This framework establishes, to the authors’ knowledge, the first parameter-level link between membrane transport and process economics across multiple draw solutions under real wastewater feed conditions.

## 2 Materials and Methods

### 2.1 Membranes and Characterization

A commercial flat-sheet cellulose-triacetate (CTA) membrane supplied by Fluid Technology Solutions (FTS H_2_O) was used throughout all experiments. Prior to each use, the membrane was stored in ultrapure water and rinsed for 20 min to remove preservatives. Intrinsic transport parameters were determined prior to the experimental campaign using standard characterization protocols. The water permeability coefficient A and the solute permeability coefficient B were obtained by RO calibration, in which the membrane was tested against a 0.5 mmol L^–1^ NaCl feed solution at applied transmembrane pressures of 0 to 20 bar, and at an ambient temperature 26 ± 1 °C. The structural parameter S was determined by operating the membrane in pressure-retarded osmosis (PRO) mode (active layer facing the draw solution). The membrane characteristics are detailed in Table 1.

**Table 1:** Characteristics of the CTA Membrane

parameter	unit	value
*A*	L m^–2^ h^–1^ bar^–1^	0.5
*B*	mol m^–2^ h^–1^	0.1
*S*	m	4·10^–4^
Membrane Area	m^2^	0.01308

### 2.2 Feed and Draw Solutions

Deionized water (DI), characterized by a conductivity below 2 μS cm^–1^, and real municipal wastewater (WW), with a chemical oxygen demand (COD) of 460 mg L^–1^ and an osmotic pressure of 2.5 bar were employed as feed solutions. The wastewater was sourced from the secondary clarifier effluent of a local municipal treatment plant, and was pre-filtered through a 0.45 μm membrane filter before use. Full physicochemical fractionation of the dissolved organic matter was outside the scope of the present study. All mechanistic interpretations relating to foulant-membrane interactions are therefore presented as the most probable explanations consistent with the available COD flux and mass-balance evidence, rather than as directly confirmed mechanisms. All experiments used wastewater from a single batch, collected on the same date from the same treatment stage, which minimizes inter-batch variability.

Draw solutions were prepared to provide an initial nominal osmotic pressure of 30 bar at 25 °C, using analytical-grade salts: NaCl (35.0 g L^–1^), MgSO_4_·7H_2_O (71.8 g L^–1^), and sodium acetate (79.4 g L^–1^). The three draw solutions were selected to represent three chemically distinct classes: a monovalent ionic solute, a divalent ionic solute, and an organic solute. This class-representative design enables systematic attribution of performance differences to specific physicochemical properties, including ion valence, hydrated radius, diffusivity, viscosity, and molecular character. Other draw solutes were excluded because they were outside the scope of the present study or incompatible with the proposed RO-based regeneration framework. Nevertheless, the proposed modeling and techno-economic framework can be readily extended to other draw solutes within the same chemical class by substituting the corresponding transport and economic parameters.

Equal initial osmotic pressure (30 bar for all three draw solutions) was chosen to isolate draw solute chemistry as the variable under study, removing osmotic driving force as a confounding factor and giving each solution the same thermodynamic starting point. Differences in resulting flux, selectivity, and fouling behavior then reflect how efficiently each solute chemistry converts that shared driving force into performance under real operating condition, so that kinetic and transport differences between solute chemistries emerge directly in the measured response.

All solutions were pre-filtered through a 0.45 μm membrane filter before use. Each of the two feed solutions (DI, WW) was systematically combined with each of the three draw solutions, yielding 6 distinct experimental configurations.

### 2.3 Experimental Setup

A rectangular cross-flow cell incorporating silicone gaskets and mesh spacers on both feed and draw sides was used to house the membrane coupon, ensuring leak-tight channels and promoting turbulence. All experiments were conducted in FO mode, with the active layer facing the feed solution (AL-FS orientation). Two variable-speed peristaltic pumps recirculated the feed (1 L) and draw (0.5 L) solutions in co-current mode at ambient temperature of 26 ± 1 °C and at a cross-flow velocity of 7.3 10^–3^ m s^–1^. The draw reservoir was placed on an analytical balance (resolution: ±0.01 g) connected to a data logger for automatic mass recording, while magnetic stirrers kept bulk homogeneity in each reservoir. Conductivity and temperature were recorded at 20-minute intervals using in-line probes.

When WW was employed as the feed solution, samples were collected hourly from both reservoirs to monitor changes in COD concentration. COD was determined following Standard Methods for the Examination of Water and Wastewater,^22^ using a closed reflux colorimetric approach with potassium dichromate as the oxidizing agent. Samples were digested at 150 °C for 120 minutes in a thermoreactor (ALB64), and absorbance was measured at 620 nm using a UV/visible spectrophotometer (Shimadzu UV-1700).

To eliminate the positive interference of chloride ions, which are present in the feed reservoir of the NaCl configurations as a consequence of reverse salt flux, mercuric sulfate (HgSO_4_) was added to each digestion vessel at a HgSO_4_:Cl^–^ mass ratio of 10:1 prior to dichromate addition, complexing chloride as the insoluble HgCl_2_ precipitate and preventing its oxidation by the dichromate reagent.

For the sodium acetate draw solution, which itself contributes to the measured COD, a blank correction was applied. The COD contribution of the draw solute at each time point was calculated from the measured conductivity-derived draw solute concentration and the theoretical oxygen demand of acetate (1.07 g COD g^–1^), and subtracted from the measured draw-side COD to isolate the contribution of permeated feed organics. No correction was required for NaCl or MgSO_4_, which register zero COD by the potassium dichromate method.

A fresh membrane coupon was used for each of the six experimental configurations to eliminate any possibility of fouling history or membrane compaction effects between runs. All coupons were taken from the same manufacturing lot to ensure consistency of intrinsic transport parameters across experiments. For each configuration, the 4 h experiment was repeated three times. Prior to each run, the membrane was flushed with DI for 20 min; the same rinse was applied post-experiment, and the coupon was stored wet until the next run.

The 4-hour experimental window was deliberately selected to capture the initial-to-intermediate fouling and concentration polarization development phases, and during which comparative draw solution performance is most clearly discriminated.^23,24^ Studies employing CTA membranes with real municipal wastewater as feed under comparable cross-flow conditions consistently report that the transition from initial cake-layer formation to advanced irreversible fouling, characterized by a measurable inflection in the flux decline curve, occurs between 8 and 24 hours of operation.^25,26^

Water flux *J*_w_ was calculated from the increase in the mass of the draw tank, while reverse solute flux *J*_s_ was determined from the feed conductivity rise using pre-established salt-specific calibration curves.^27^

1 $$J_{w}=\frac{\Delta V}{A_{m}\times \Delta t}$$

2 $$J_{s}=\frac{C_{t}\times V_{t}-C_{0}{\times V}_{0}}{A_{m}\times \Delta t}$$

The conductivity–concentration calibration was established from standard solutions over the full concentration range encountered in the draw reservoir during experiments, yielding a well-defined monotonic relationship (*R* ^2^ = 0.997). In the case of sodium acetate, while conductivity in the draw reservoir reflects the contributions of all ionic species present, the electroneutrality constraint governing co-diffusion of Na^+^ and CH_3_COO^–^ through the dense CTA active layer maintains the ionic ratio in the draw solution to a reasonable approximation, preserving the validity of the conductivity-based concentration estimate. This represents a bounded source of uncertainty in the *J*_s_ estimate for sodium acetate that is acknowledged as a limitation of the conductivity-based approach.

The ratio of the reverse salt flux to the water flux defines the specific reverse salt flux *J*_sw_ (mol L^–1^). This ratio serves as a system-level performance indicator of membrane selectivity under the prevailing operating conditions, reflecting the combined influence of membrane solute permeability, draw solution chemistry, and concentration polarization. A lower *J*_sw_ signifies a more selective membrane, indicating a relatively higher transport of water compared to the draw solute, independently of the absolute magnitude of the individual transmembrane fluxes.

The COD solute flux from the feed to the draw solution was determined based on the change in COD mass in the draw solution over time, normalized by the effective membrane area.

3 $$J_{COD}=\frac{{COD}_{D,t}{\times V}_{t}-{COD}_{D,0}{\times V}_{0}}{A_{m}\times \Delta t}$$

The specific COD flux, defined as the ratio *J*_COD_/*J*_w_ (mg L^–1^), normalizes organic matter permeation by the water driving force and provides a measure of selectivity decline independent of flux magnitude.

To further characterize the performance of FO when treating wastewater (WW) as the feed solution, several key performance indicators were assessed, namely the volume concentration factor (CF_V_) (eq 4), COD concentration factor (CF_COD_) (eq 5), COD rejection rate (*R*) (eq 6), water recovery (WR) (eq 7), and the accumulated mass of foulants (*M*_foul_) (eq 8).

4 $$CF_{v}=\frac{VF_{0}}{VF_{t}}$$

5 $${\mathrm{CF}}_{COD}=\frac{{\mathrm{COD}}_{F,t}}{\mathrm{CO}D_{F,0}}$$

6 $$R=\left(1-\frac{{\mathrm{COD}}_{D}}{{\mathrm{COD}}_{F}}\right)\times 100$$

7 $$\mathrm{WR}=\left(VF_{0}-VF_{t}\right)/VF_{0}\times 100$$

8 $$M_{\mathrm{foul}}=\left(\mathrm{CO}D_{F,0}\times VF_{0}+{\mathrm{COD}}_{D,0}\times VD_{0}\right)-\left(\mathrm{CO}D_{F,t}\times VF_{t}+{\mathrm{COD}}_{D,t}\times VD_{t}\right)$$

The reduction in solution volume due to sampling was accounted for in all equations. All trials were performed in triplicate. The combined measurement uncertainty for each reported flux variable was estimated by propagating all independent error sources, including instrument resolution, probe accuracy, temperature deviation, cross-flow velocity, and inter-run reproducibility. The coefficient of variation on *J*_w_, *J*_s_, and *J*_COD_ was consistently below 1%, 3.5%, and 3%, respectively.

## 3 FO Modeling

### 3.1 Theoretical Framework

Water flux in FO processes is described by the following equation^28^

9 $$J_{w}=A\times {\Delta \pi }_{bulk}$$

Where *J*_w_ (L m^–2^ h^–1^), A (L m^–2^ h^–1^ bar^–1^), and Δπ_bulk_ (bar) are the water flux, the intrinsic water permeability, and the bulk osmotic pressure difference across the membrane, respectively.

Equation 9 provides an idealized approximation that does not consider ICP, ECP, or RSD, phenomena that substantially reduce the effective osmotic driving force across real asymmetric membranes. To account for this coupled resistance, and assuming the AL-FS orientation described in Section 2.3, the water flux can be more accurately expressed as^28^

10 $$J_{w}=A\times \left(\pi_{i}-\pi_{Fm}\right)$$

Where π_i_ and π_Fm_ represent the osmotic pressures at the selective layer/support layer and selective layer/feed solution interfaces, respectively. These interfacial pressures are determined from the solute concentrations at the active-layer surface (*C*_Fm_), support-layer surface (*C*_Dm_), and support/active-layer interface (*C*_i_). They are calculated by assuming one-dimensional, steady-state transport and integrating the convection–diffusion equation across the boundary layer (film theory) and support layer (ICP model). The resulting interfacial concentrations are given by^29,30^

11 $$C_{Dm}=C_{Db}\times \exp\left(\frac{{-J}_{w}}{k_{D}}\right)-\frac{J_{s}}{J_{w}}\times \left[1-\exp\left(\frac{{-J}_{w}}{k_{D}}\right)\right]$$

12 $$C_{Fm}=C_{Fb}\times \exp\left(\frac{J_{w}}{k_{F}}\right)+\frac{J_{s}}{J_{w}}\times \left[\exp\left(\frac{J_{w}}{k_{F}}\right)-1\right]$$

13 $$C_{i}=C_{Dm}\times \exp\left(-J_{w}\frac{S}{D}\right)-\frac{J_{s}}{J_{w}}\times \left[1-\exp\left(J_{w}\frac{S}{D}\right)\right]$$

Here, *C*_Fb_ and *C*_Db_ (mol m^–3^) are the bulk solute concentrations in the feed and draw solutions. δ_F_ and δ_D_ (m) are the boundary-layer thicknesses, and *k*_F_ and *k*_D_ (m s^–1^) are the mass-transfer coefficients. *D* (m^2^ s^–1^) is the diffusion coefficient within the porous support. *S* (m) denotes the membrane structural parameter, defined below (eq 14) as a function of the membrane tortuosity τ (−), porosity ε (−), and the thickness of the support layer *t*_s_ (m)^8,30^

14 $$S=\frac{t_{s}\times \tau }{\varepsilon }$$

The numerical value of *S* used in this study is reported in Table 1 (Section 2.1) and was determined by pressure-retarded osmosis (PRO) mode matching. The mass transfer coefficient, on each side of the membrane, is determined using the following equation

15 $$k=\frac{\mathrm{Sh}{\times D}_{\propto }}{d_{h}}$$

Where *Sh* (−), *d*_h_ (m), and *D*_∝_ (m^2^ s^–1^) are the Sherwood number, the hydraulic diameter, and the bulk diffusion coefficient, respectively. *Sh* is determined according to the flow regime. It is calculated as^29^

For laminar conditions (*Re* < 2100)

16 Sh=1.85×(Re×Sc×dhL⁡)0.33

For turbulent conditions (*Re* > 2100)

17 Sh=0.04×Re0.75×Sc0.33

Where *Re* (−), *Sc* (−), and *L* (m) are respectively the Reynolds number, the Schmidt number, and the channel length.

With respect to solute transport, the reverse salt flux *J*_s_ (mol m^–2^ h^–1^) is proportional to the membrane’s solute permeability *B* (L m^–2^ h^–1^) and to the salt-concentration difference across the active layer, as given by^28^

18 $$J_{s}=B\times \left(C_{i}-C_{Fm}\right)$$

when real wastewater is employed as the feed solution, the transport phenomena at the membrane interface extend beyond the classical water flux and reverse salt flux framework. They also encompass convective permeation of organic matter and the progressive accumulation of foulants on the membrane active layer. The flux of chemical oxygen demand across the membrane, *J*_COD_ (mg m^–2^ h^–1^), governs the transfer of dissolved organic compounds from the feed to the draw solution side and is expressed as^31^

19 $$J_{\mathrm{CO}D}=P_{COD}\times \left({COD}_{F}-{COD}_{D}\right)$$

Where *P*_COD_ (L m^–2^ h^–1^) is the COD transfer coefficient, representing the effective membrane permeability to the dissolved organic solutes. COD_F_ and COD_D_ (mg L^–1^) are the COD concentrations in the feed and draw solutions, respectively.

The purely adopted diffusive formulation of *J*_COD_ omits the convective solute-drag term, an approximation whose validity was assessed by estimating the membrane-scale Péclet number for COD transport across the cellulose-triacetate (CTA) membrane active layer. The Péclet number is defined as *Pe* = *J*_w_δ/*D*_COD_. Where δ ≈ 1 × 10^–6^ m is the CTA active layer thickness and *D*_COD_ ≈ 5 × 10^–10^ m^2^ s^–1^ is the effective diffusion coefficient for low-molecular weight dissolved organics, consistent with values reported in the forward osmosis organic transport literature.^32^ Assuming a maximum water flux of 5 L m^–2^ h^–1^, the resulting Péclet number was estimated to be 0.0025 ≪ 1. Thus, diffusion is overwhelmingly the dominant transport mechanism and the convective contribution to total COD flux is less than 0.2% under all tested conditions. The diffusion-only approximation is therefore physically justified across the full range of fluxes encountered in the present study, and its adoption introduces no meaningful error in the COD transport predictions.

In parallel with solute permeation, the presence of complex organic matrices in real wastewater feeds induces membrane fouling through the convective deposition of suspended and colloidal matter onto the active layer surface.^33^ The fouling flux *J*_foul_ (mg m^–2^ h^–1^) is expressed as a function of feed organic loading

20 $$J_{foul}=K_{foul}\times {\mathrm{CO}D}_{F}$$

Where *K*_foul_ (L m^–2^ h^–1^) is the fouling coefficient, representing the rate of the effective foulant deposition. The assumption of the first order fouling was established in Section 2.3, and will be confirmed experimentally for all six DS/FS configurations by the absence of a measurable inflection point in the flux decline trajectories (Section 5.1.1).

It is important to distinguish the diffusion-dominated transmembrane transport of dissolved organic solutes, governed by *Pe* ≪ 1 at the active layer scale, from the convective deposition of suspended and colloidal foulants onto the membrane surface, which is governed by permeate-flux-driven transport across the external hydrodynamic boundary layer. The two processes operate at fundamentally different length scales: the active layer thickness (δ ≈ 1 × 10^–6^ m) for dissolved organic permeation, and the hydrodynamic boundary layer thickness (δ_BL_ ≈ 10^–4^ to 10^–3^ m) for foulant convection. They also involve distinct classes of organic matter, dissolved low-molecular-weight species in the former case, and particulate and colloidal matter in the latter, for which diffusion across the boundary layer is negligible on experimental timescales. Therefore, the diffusion-based Péclet criterion for organic leakage does not apply to the convective foulant deposition term *J*_foul_.

The instantaneous COD concentrations in each compartment are defined as

21 $${\mathrm{CO}D}_{D}=\frac{M_{{COD}_{D}}}{V_{D}}$$

22 $${\mathrm{CO}D}_{F}=\frac{M_{{COD}_{F}}}{V_{F}}$$

Where *M*_CODF_ and *M*_CODD_ (mg) are the COD mass in the feed and draw solutions. *V*_F_ and *V*_D_ (L) are the volume of feed and draw solutions.

The dynamic mass balances governing the evolution of COD across all three compartments are written as

23 $$\frac{dM_{{\mathrm{CO}D}_{F}}}{dt}=-A_{m}\times J_{COD}-A_{m}{\times J}_{foul}$$

24 $$\frac{dM_{{\mathrm{CO}D}_{D}}}{dt}=A_{m}\times J_{COD}$$

25 $$\frac{dM_{{\mathrm{CO}D}_{foul}}}{dt}=A_{m}{\times J}_{foul}$$

This system of equations satisfies global COD conservation at all times

26 $$M_{{\mathrm{CO}D}_{0}}=M_{{COD}_{F}}\left(t\right)+M_{{COD}_{D}}\left(t\right)+M_{{COD}_{foul}}\left(t\right)$$

Where *M*_COD0_ is the total initial COD mass in the system. This conservation condition provides a physically consistent closure to the theoretical framework and ensures that the three mechanisms, concentration, leakage, and fouling, collectively and exclusively account for the total initial COD mass in the system, satisfying global mass conservation at all times. The partition between the leakage and the fouling terms is nevertheless subject to model uncertainty: any dissolved COD that adsorbs reversibly to the membrane surface and desorbs during sampling would be attributed to the fouling term.

### 3.2 Numerical Simulations

An explicit finite-difference scheme was implemented in MATLAB under conditions identical to those of the experiments. Figure 1 provides a detailed flowchart of the FO process modeling. After parameter initialization, the variables of the feed and draw solutions were computed sequentially.

**Figure 1:**
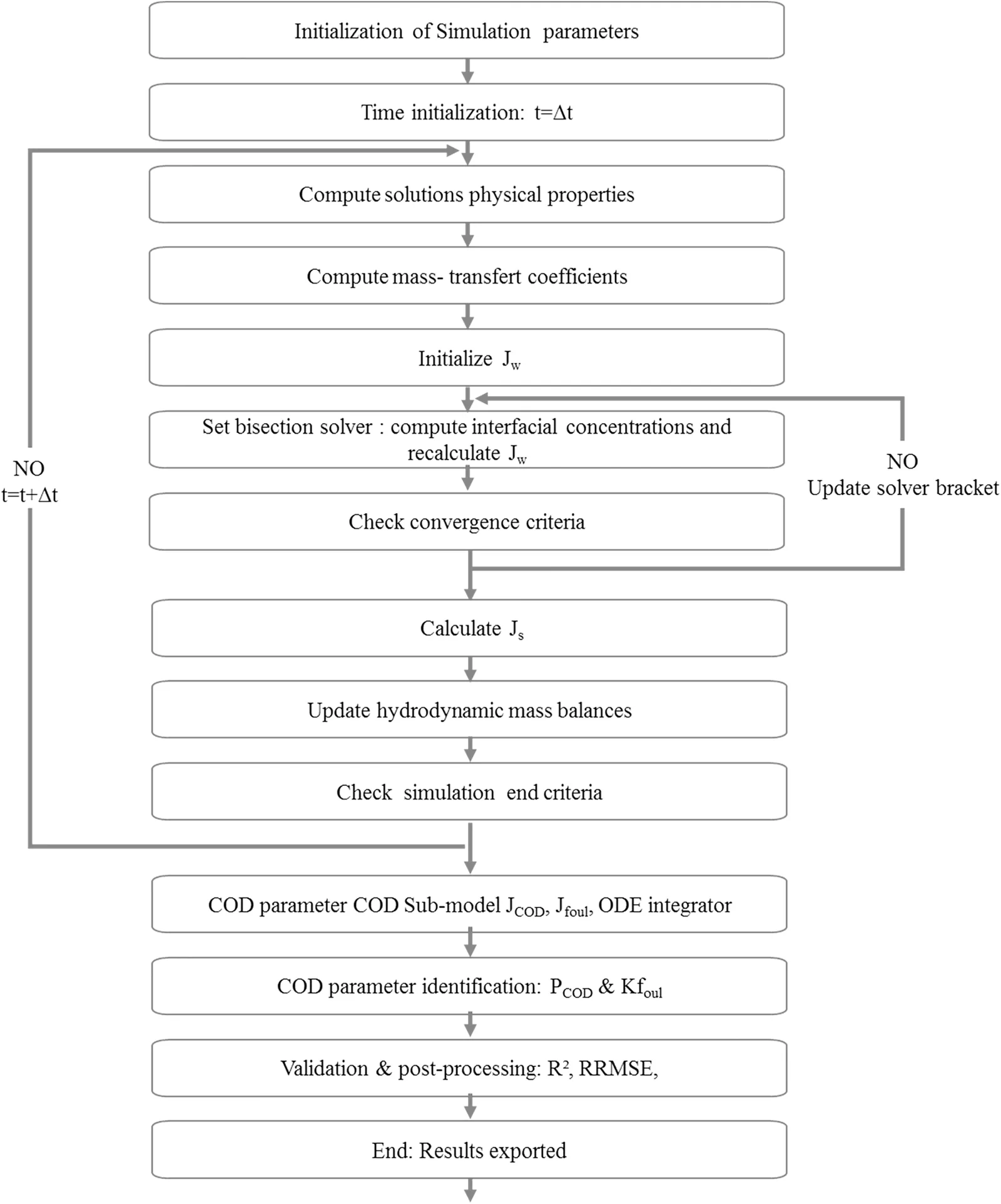
Flowchart of the FO process-modeling framework.

The interfacial concentrations were applied under a quasi-steady-state assumption using a time step chosen so that the profiles equilibrate almost instantaneously relative to changes in bulk concentration. The water flux *J*_w_ was then estimated using a bisection solver iterated to within 1% absolute error. With the overall mass-balance equations, concentrations, volumes, and physical properties were refreshed at each time step until the final experimental time was reached. Solution properties were estimated using empirical equations depending on solution concentration and temperature.^34^ At each time step, the model yields the reverse solute flux *J*_s_, as well as the full concentration field across the membrane, providing a spatially resolved description of solute distribution throughout the membrane system.

The quality of the model was evaluated through the coefficient of determination *R* ^2^ and the root mean square error (RMSE), relative RMSE (RRMSE), and maximum relative error (MRE), computed between simulated and experimental profiles.

A COD sub-model was embedded within this finite-difference framework, with the temporal evolution of *V*_F_ and *V*_D_ supplied directly from the hydrodynamic solver outputs at each time step, thereby decoupling organic transport estimation from osmotic pressure computations for the complex wastewater matrix.^35^

COD temporal evolution was governed by three distinct mechanisms: (i) apparent concentration of COD in the feed solution resulting from continuous water extraction across the membrane; (ii) transmembrane leakage of dissolved organic from the feed solution (FS) to the draw solution (DS) driven by the transmembrane concentration gradient; and (iii) irreversible removal of COD from the liquid phase due to membrane fouling and adsorption onto the membrane surface or within its porous structure. The following simplifying assumptions were adopted:

- COD behaves as a lumped scalar parameter integrating all dissolved organic species grounded in two facts. Firstly, measurable shifts in organic molecular-weight distributions are unlikely over the 4-h experimental period. Secondly, the dense CTA active layer restricts permeation to a specific low-molecular-weight fraction, represented by the effective parameter *P*_COD_;
- Both FS and DS bulk reservoirs are assumed to be perfectly mixed in the bulk reservoir at all times;
- membrane properties and operating temperature remain constant throughout the experiment;
- COD transport across the membrane is governed by an apparent diffusion mechanism;
- Fouling kinetics follow a first-order dependence on feed-side COD concentration, consistent with the initial-to-intermediate fouling regime established in Section 2.3.

The coupled ordinary differential equation (ODE) system (eqs 23–25) was integrated using the built-in MATLAB ODE solver, and the global COD conservation condition (eq 26) was enforced as an internal numerical stability diagnostic at each iteration.

The two model parameters, *P*_COD_ and *K*_foul_, were identified through a numerical calibration procedure designed to minimize the discrepancy between simulated and experimentally observed COD concentrations in both the feed and draw solutions over time. The identification was carried out by systematically evaluating the sum of squared residuals (SSR) between model outputs and experimental measurements across the admissible parameter space, selecting the parameter pair that yields the global minimum of SSR. The admissible parameter space was established using physically meaningful ranges drawn from the FO organic transport literature: *P*_COD_ in [0.005, 0.5] L m^–2^ h^–1^ and *K*_foul_ in [0.005, 0.5] L m^–2^ h^–1^.^6,33,36^

To assess parameter identifiability, the SSR surface was evaluated over the full admissible [*P*_COD_, *K*_foul_] parameter space which was dilated by 10% on either side. For all three draw solutions, the SSR landscape exhibited a single well-defined global minimum with no evidence of a correlation ridge between *P*_COD_ and *K*_foul_, confirming that the two parameters are independently identifiable from the available COD data in both compartments. It should be noted that *P*_COD_ and *K*_foul_ are empirical, membrane- and feed-specific calibration parameters rather than intrinsic membrane transport coefficients. Their absolute values are expected to vary with active-layer chemistry, support-layer structure, and feed organic composition.

The quality of the calibration was evaluated through the coefficient of determination *R* ^2^, the root mean square error (RMSE), mean absolute error (MAE), and mean bias error (MBE), computed between simulated and experimental COD profiles, ensuring that the identified parameter set provides a statistically robust and physically consistent representation of organic transport and fouling dynamics under real wastewater operating conditions.

## 4 Techno-Economic Analysis Framework

### 4.1 Levelized Cost of Water

To provide an economically grounded evaluation of the three draw solutions, a dedicated techno-economic framework was developed to assess the Levelized Cost of Water (LCOW) for each configuration when using WW as FS. The economic model is calibrated directly against experimentally measured water flux and reverse salt flux values, establishing a quantitative link between membrane transport performance and process lifecycle cost. The assessment is based on a hypothetical plant with a target water production capacity (*Q*_p_) of 1000 m^3^ day^–1^ operating over a project lifetime (T) of 20 years.^15^ The economic model systematically accounted for both Capital Expenditure (CAPEX) and Operational Expenditure (OPEX), with a discount rate (*r*_d_) of 8% applied for financial calculations using^16^

27 $$\mathrm{LCOW}=\frac{\mathrm{CAPEX}\times \mathrm{CRF}+\mathrm{OPEX}}{Q_{p}}$$

where CRF is the Capital Recovery Factor calculated as^16^

28 $$CRF=\frac{r_{d}\times {\left(1+r_{d}\right)}^{T}}{{\left(1+r_{d}\right)}^{T}-1}$$

CAPEX for each configuration was determined from the cost of FO membrane modules (CAPEX_Memb_) and the Reverse Osmosis (RO) regeneration unit (CAPEX_RO_). Feed pretreatment costs are excluded from the system boundary and should be added for site-specific economic evaluation. RO was adopted as a unified regeneration basis for all three draw solutions, enabling direct comparison against a common cost reference and reflecting the thermodynamic feasibility of RO regeneration at the osmotic pressures tested.

A unit membrane cost of USD 50 m^–2^ was applied, reflecting current industrial benchmarks for high-performance FO membranes.^15^ The required membrane area Am, for the FO process was calculated as^21^

29 $$A_{m}=\frac{Q_{p}}{t_{op}\times J_{w}}$$

Where *t*_op_ is the number of operational hours per day (24 h for continuous operation).

The CAPEX for the RO regeneration unit was estimated using a first-order approximation, by scaling a baseline cost (*C*_RO,base_) of USD 1 m^–3^ day^–1^, typical for seawater RO plants, according to the initial osmotic pressure of the specific draw solution.^37,38^ A scaling factor was accounted for the increased capital investment required for RO systems operating against higher osmotic pressures. The CAPEX_RO_ is given by^37^

30 $${\mathrm{CAPEX}}_{\mathrm{RO}}=C_{\mathrm{RO},\mathrm{base}}\times Q_{p}\times \frac{{\Delta \pi }_{\mathrm{DS}}}{{\Delta \pi }_{\mathrm{SW}}}$$

This linear scaling is consistent with observations that RO capital cost increases proportionally with required operating pressure at fixed plant capacity and is adopted here as a first-order approximation. Its validity is supported by the fact that all three draw solutions were prepared at identical initial osmotic pressures approximating that of seawater. Thus, all three configurations were placed within the same engineering cost bracket as the seawater RO baseline and the systematic bias introduced by the linear assumption was limited to within the uncertainty range of the baseline cost itself. Other ancillary equipment costs were implicitly included within these primary component estimations for comparative purposes.

OPEX included energy consumption, draw solute replenishment, membrane replacement and maintenance, and membrane cleaning. Energy cost, OPEX_energy_ was calculated based on an assumed electricity price (*C*_e_) of USD 0.10 kWh^–1^. The specific energy consumption (SEC) for the RO regeneration unit, SEC_RO_, was modeled as a function of the draw solution’s osmotic pressure, Δπ_DS_, assuming an applied pressure 1.2 times the osmotic pressure and a pump efficiency of 75%. An additional 0.5 kWh m^–3^ was allocated to FO pumping (SEC_FO_).

31 $${O\mathrm{PE}X}_{energy}=\left({SEC}_{RO}+S{EC}_{FO}\right)\times Q_{p}\times C_{e}\times H_{op}$$

Where *H*_op_ represented annual operating hours (8000 h/year).

Draw solute replenishment cost (OPEX_solute_) was determined by the average experimental reverse salt flux, the total membrane area (*A*_m_), annual operating hours (*H*_op_), and the specific market price of each draw solute (*C*_DS_ )^21^

32 $${O\mathrm{PE}X}_{solute}=J_{s}\times A_{m}\times H_{op}\times C_{DS}$$

Membrane replacement cost OPEX_RM_ was obtained by dividing the membrane CAPEX by the assumed membrane service life, which was considered to be 5 years. The annualized cleaning cost (OPEX_Clean_) was calculated as the sum of 3 operational components representing the principal cost drivers in membrane fouling management^39^

33 $${O\mathrm{PE}X}_{Clean}=N_{clean}\times \left(C_{chem}+C_{energy}+C_{downtime}\right)$$

Where *N*_clean_, *C*_chem_, *C*_energy_, and *C*_downtime_ were the annual number of cleaning events, chemical reagent costs, pumping and thermal energy costs, and production downtime costs, respectively.

Chemical costs for membrane cleaning were estimated as the product of cleaning solution volume, cleaning agent concentration, and unit price of cleaning chemicals. For organic fouling removal, sodium hydroxide (NaOH) was applied at 0.1 wt % concentration with a cleaning solution volume of 5 L m^–2^.^36^

Energy costs were associated with hydraulic pumping and thermal heating of cleaning solutions. Heating requirements were based on a target cleaning in place (CIP) temperature of 40 °C.^39,40^ The annual cleaning frequency was calculated from the duration of each cleaning event and the operating time required to reach the cleaning trigger. The cleaning trigger was defined as the time required for the transmembrane water flux to decline by a threshold fraction α = 15% of the initial flux *J*_w0_, in accordance with standard membrane operation practice.^41^

To project laboratory-derived cleaning intervals to industrial conditions, the operating time between cleanings was scaled by a factor defined as the ratio of the typical industrial solution velocity (0.2 m s^–1^) to the experimental velocity.^42–44^ The velocity ratio scaling applied here is a first-order approximation whose accuracy depends on the assumption that fouling kinetics under industrial cross-flow conditions scales linearly with wall shear rate relative to the laboratory configuration. To assess the economic sensitivity of this extrapolation, α was varied between severe fouling trigger of 5% to a reduced fouling trigger of 25%.

Production downtime costs represented the economic value of lost permeate during each cleaning event that lasts 3 hours.^45,46^ Annual maintenance and labor costs, OPEX_M&L_, were estimated as 5% of the total CAPEX.^17^

### 4.2 Uncertainty Quantification and Sensitivity Analysis

The deterministic LCOW framework yields point estimates that are conditioned on the mean experimental flux values but do not account for the variability inherent in triplicate laboratory measurements. To address this limitation, a Monte Carlo uncertainty propagation procedure was implemented to translate experimental flux uncertainty directly into a probability distribution of LCOW for each draw solution configuration.

At each of *N* = 10,000 iterations, the time-averaged water flux *J*_w_ and reverse salt flux *J*_s_ were independently sampled from normal distributions defined by their experimentally measured means and standard deviations across triplicate runs. All remaining techno-economic parameters were held at their nominal values, isolating the contribution of flux measurement uncertainty to total LCOW uncertainty. Non-physical samples were prevented by enforcing a lower bound of 0.01 L m^–2^ h^–1^ on sampled flux values, a constraint that was never active under the present experimental conditions. A fixed random seed was applied to ensure reproducibility of the reported results.

For each sampled flux pair, the full LCOW calculation was executed. The resulting distribution of 10,000 LCOW values was used to report, alongside each deterministic point estimate, the mean, standard deviation, and 95% confidence interval (CI) defined by the 2.5th and 97.5th percentiles of the distribution.

In addition, a one-at-a-time sensitivity analysis was conducted to quantify the individual response of LCOW to variation in four techno-economic parameters: membrane unit cost (USD 10–80 m^–2^), water flux (1–15 L m^–2^ h^–1^), discount rate (2–16%), and electricity price (USD 0.05–0.35 kWh^–1^). Each parameter was varied independently across its defined range while all others were fixed at their nominal values, yielding a set of LCOW response curves that identify the parameters to which each draw solution configuration is most sensitive and quantify the magnitude of LCOW variation attributable to each. The one-at-a-time approach varies each parameter independently and therefore does not capture interaction effects between correlated parameters, such as the covariation of discount rate and electricity price. This limitation should be considered when interpreting the magnitude of the sensitivity gradients reported in Section 5.4.2.

The input parameters, assumed values, and their corresponding sources or modeling assumptions used to compute the levelized cost of water (LCOW) for each draw solution are summarized in Table 2.

**Table 2:** Input Parameters and Assumptions Used in the Techno-Economic (LCOW) Analysis

parameter	value	source
Target water production capacity	1000 m^3^ day^–1^	Assumption, ref 15
Project lifetime	20 years	Assumption, ref 15
Annual operating hours	8000 h year^–1^	Assumption
Discount rate	8%	Assumption, ref 16
Unit FO membrane cost	USD 50 m^–2^	Industrial benchmark, ref 15
Baseline RO capital cost	USD 1 (m^3^ day^–1^)^−1^	refs 37,38
Electricity price	USD 0.10 kWh^–1^	Assumption
RO applied pressure factor	1.2 × osmotic pressure	Assumption
RO pump efficiency	75%	Assumption
FO pumping specific energy consumption	0.5 kWh m^–3^	Assumption
NaCl solute unit market price	USD 0.1 kg^–1^	Ref 21
MgSO_4_ solute unit market price	USD 0.3 kg^–1^	Ref 21
Sodium acetate solute unit market price	USD 0.8 kg^–1^	Ref 21
Membrane service life	5 years	Assumption
Annual maintenance & labor cost	5% of total CAPEX	Assumption, ref 17
Cleaning agent (NaOH) concentration	0.1 wt %	Ref 36
Cleaning solution volume	5 L m^–2^	Ref 36
CIP (clean-in-place) temperature	40 °C	Refs 39,40
Cleaning trigger	15% of initial flux *J*_w_	refs 41
Cleaning trigger sensitivity range	5% (severe) to 25% (reduced)	Sensitivity scenario
Industrial velocity	0.2 m s^–1^	refs 42–44
Duration of each cleaning event	3 h	refs 45,46
Sensitivity range: membrane unit cost	USD 10–80 m^–2^	Sensitivity scenario
Sensitivity range: water flux	1–15 L m^–2^ h^–1^	Sensitivity scenario
Sensitivity range: discount rate	2–16%	Sensitivity scenario
Sensitivity range: electricity price	USD 0.05–0.35 kWh^–1^	Sensitivity scenario

## 5 Results and Discussion

### 5.1 Evolution of Fluxes over Time

To evaluate the influence of feed and draw solution characteristics on FO performance, experiments were carried out using two types of feed solutions (DI and WW) and three draw solutions (NaCl, MgSO_4_, and sodium acetate). For each combination, the temporal evolution of water flux (*J*_w_), salt flux (*J*_s_), specific reverse flux, and COD flux (*J*_COD_) was monitored and compared to model predictions, as shown in Figures 2, 3, 4 and 5.

**Figure 2:**
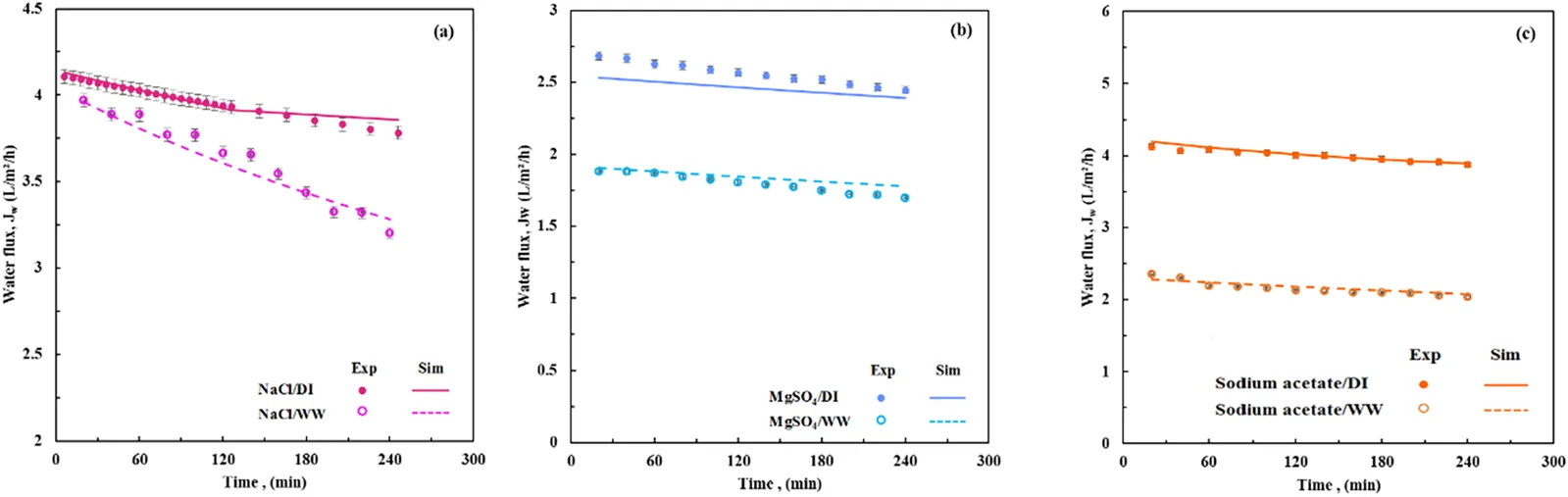
Experimental and predicted temporal profile of water flux for the three DS (NaCl (a), MgSO_4_ (b), and sodium acetate (c)), using DI­(filled symbols for experimental means, solid lines for model predictions) and WW (open symbols for experimental means, dashed lines for model predictions) as FS.

**Figure 3:**
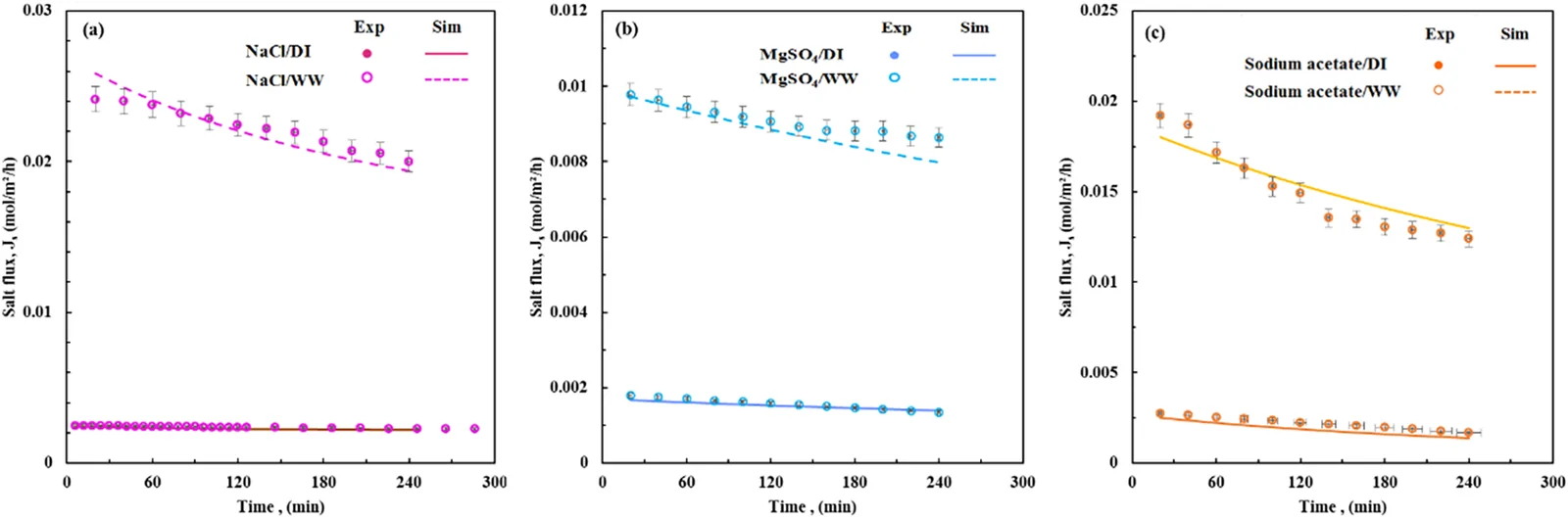
Experimental and predicted temporal profile of salt flux for the three draw solutions­(NaCl (a), MgSO_4_ (b), and sodium acetate (c)), using DI­(filled symbols for experimental means, solid lines for model predictions) and WW (open symbols for experimental means, dashed lines for model predictions) as FS.

**Figure 4:**
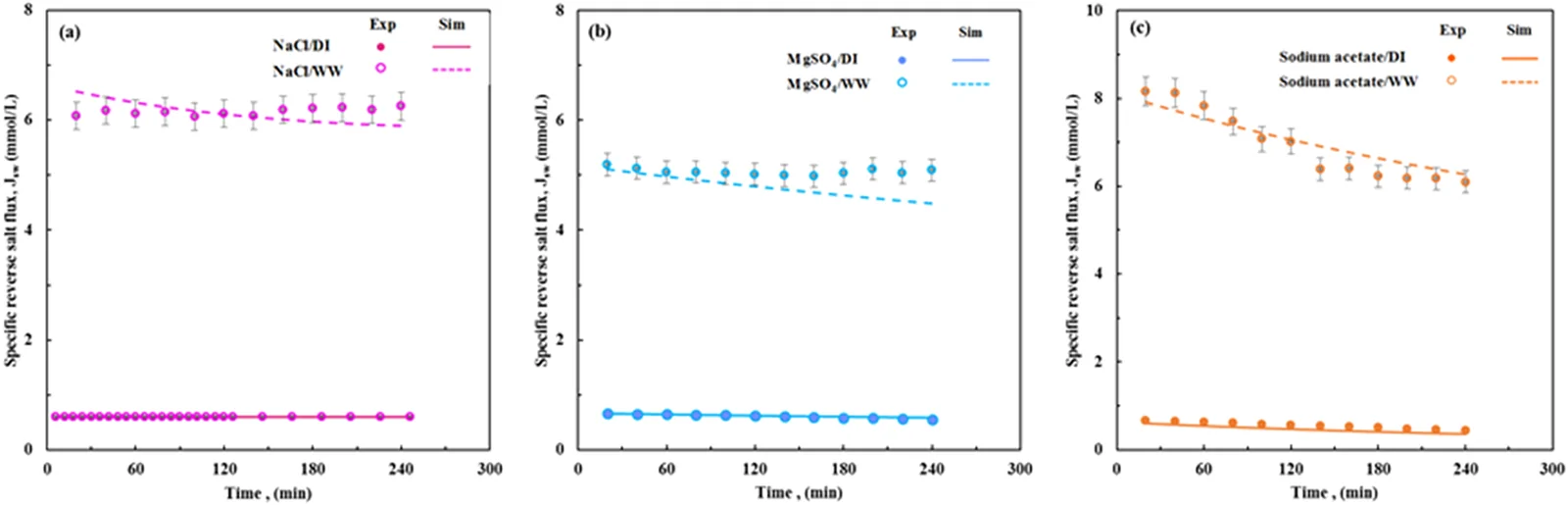
Experimental and predicted temporal profile of *J*_sw_ for the three DS (NaCl (a), MgSO_4_ (b), and sodium acetate (c)), using DI­(filled symbols for experimental means, solid lines for model predictions) and WW (open symbols for experimental means, dashed lines for model predictions) as FS.

**Figure 5:**
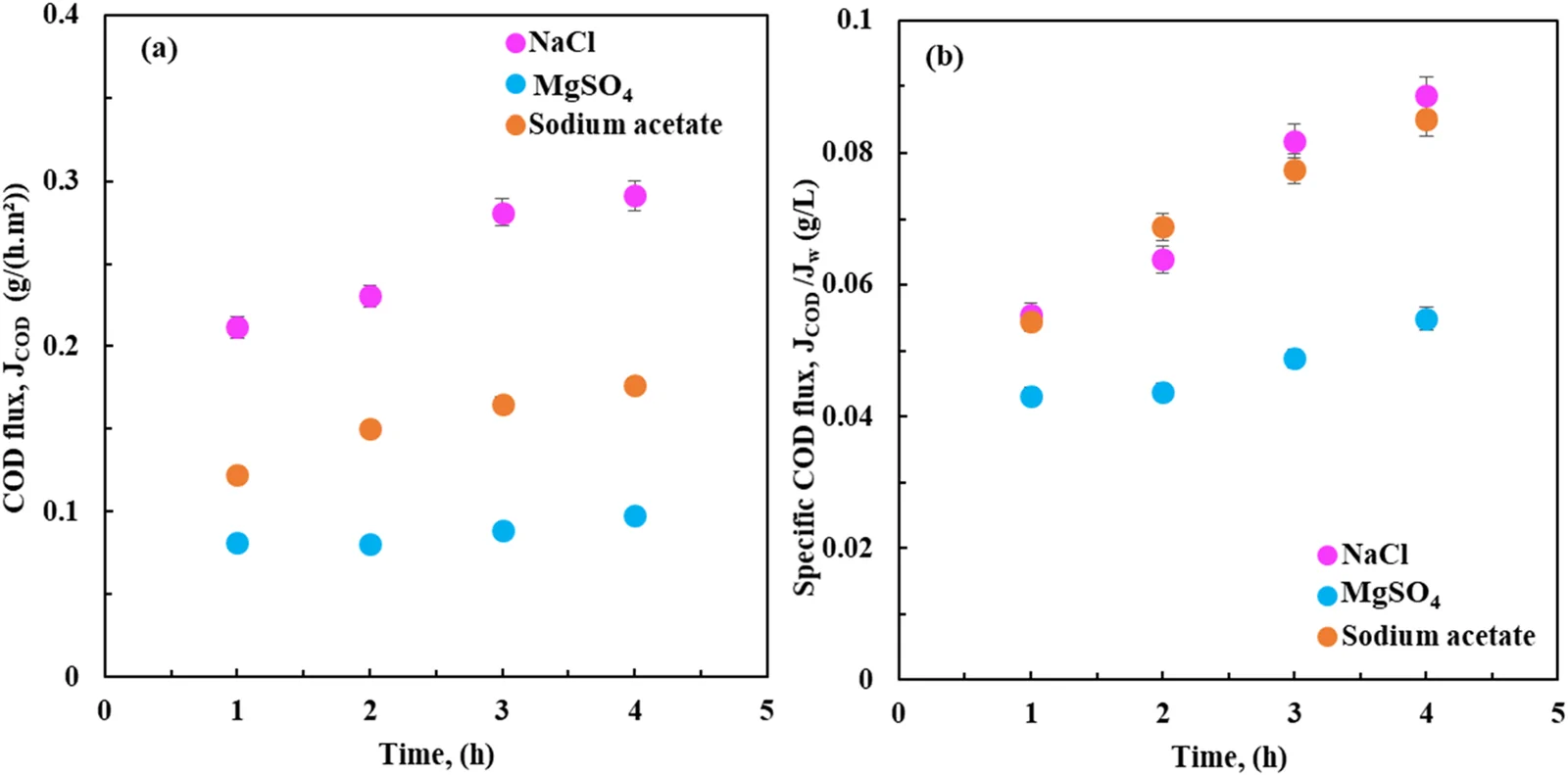
Temporal evolution of (a) COD solute flux (*J*_COD_), and (b) COD specific flux.

#### 5.1.1 Water Flux (*J*_w_) Evolution

Figure 2 shows the temporal variation of *J*_w_ for the three draw solutions DS (NaCl (a), MgSO_4_ (b), and sodium acetate (c)) under DI and WW conditions. A gradual decrease in flux was observed for all DS/FS combinations over the 4-hour experimental period, reflecting the progressive development of concentration polarization (CP) and initial foulant accumulation at the active layer surface, consistent with classical FO behavior in the initial-to-intermediate fouling regime. The quantitative contributions of each CP mechanism to total osmotic driving force attenuation are presented in Section 5.3.

A higher initial water flux was observed when DI water was used as the feed solution, owing to the larger osmotic pressure difference between the feed and draw sides. The use of WW, a complex matrix containing dissolved salts and organic matter, generated a lower *J*_w_. This decline is consistent with two non-exclusive mechanisms: a reduction in the effective osmotic driving force caused by feed-side solutes, and an increase in hydraulic resistance from external foulant deposition at the membrane active layer. Direct membrane surface characterization was outside the scope of the present study and would be required to apportion the relative contribution of each mechanism with certainty.

As a result of differences in the ionic properties and osmotic behavior of the respective draw solutions, the transition from DI to WW feed led to an average decrease in *J*_w_ by 8.14%, 29.87%, and 46.19% for NaCl, MgSO_4_, and sodium acetate, respectively.

For NaCl, a draw solute composed of monovalent ions, the decrease was minimal because these ions exhibit high diffusivity and mobility that limit CP and sustain a strong osmotic driving force.^47^ For MgSO_4_, a multivalent ionic draw solution, the decline was more pronounced due to lower ion diffusivity, larger hydrated radii, and stronger electrostatic interactions, which intensify CP and reduce the effective osmotic pressure.^48,49^ For the organic draw solutes, sodium acetate, the flux reduction was the most severe. This behavior is most consistent with elevated solution viscosity, which increases hydraulic resistance to water permeation, and with the possibility of hydrophobic interactions between the organic draw solute and the CTA active layer, which could further reduce surface water affinity.^50,51^

Three indicators were computed and summarized in Table 3 to quantify the rate of decay. The first indicator is the overall decay rate k (h^–1^) defined as the exponential decay constant fitted over the entire 4-h run. The second is the initial decay rate *k*_0_ (h^–1^), representing the instantaneous rate of flux decline at *t* = 0 h. The third indicator is the relative cumulative loss RCL (%), defined as the percentage difference between the time-averaged flux and the initial flux, providing an integrated measure of the total permeate loss over the experiment.

**Table 3:** Flux Decay Parameters (*k*, *k*_0_, RCL) for All DS/FS Combinations

DS	FS	*k* (h^–1^)	*k*_0_ (h^–1^)	RCL (%)
NaCl	DI	0.021	0.025	4.28
	WW	0.058	0.067	8.84
MgSO_4_	DI	0.025	0.032	4.55
	WW	0.03	0.046	4.5
Sodium acetate	DI	0.016	0.021	3.01
	WW	0.034	0.078	8.68

According to Table 3, the highest absolute decay rate *k* and the largest cumulative flux loss RCL were observed for the WW/NaCl combination (*k* = 0.058 h^–1^, RCL = 8.84%), while the lowest values were recorded for DI/sodium acetate (*k* = 0.016 h^–1^, RCL = 3.01%). Switching from DI to WW consistently accelerated flux decline across all draw solutions, as evidenced by increases in all three decay metrics, with markedly different magnitudes. For NaCl and sodium acetate, the decay rate k and the initial decay rate *k*_0_ more than doubled (*k*: 0.021 to 0.058 and 0.016 to 0.034 h^–1^, respectively), indicating that the organic and ionic content of real wastewater imposed a strong additional resistance through early fouling layer formation and enhanced ICP within the 4-h observation window. In contrast, for MgSO_4_, the decay rate remained nearly unchanged (*k*: 0.025 to 0.030 h^–1^) and the RCL was stable (4.55 to 4.50%).

The experimental water flux profiles corroborate the adequacy of the selected 4-h experimental window. For all six DS/FS configurations, *J*_w_ exhibited a smooth, monotonically declining trajectory throughout the duration of the experiment, with no measurable inflection point indicative of a transition to advanced fouling regimes. The absence of acceleration followed by abrupt stabilization of the flux decline rate confirmed that the fouling regime had not transitioned to advanced compaction or irreversible deposition within the observation window (Section 2.3).

#### 5.1.2 Salt Flux (*J*_s_) Evolution

Figure 3 presents the experimental and predicted temporal profile of salt flux for all configurations. Under DI as FS, the measured salt fluxes followed the order *J*_s_ (NaCl ) > *J*_s_ (sodium acetate) > *J*_s_ (MgSO_4_), with *J*_s_ (NaCl) exceeding *J*_s_ (sodium acetate) and *J*_s_ (MgSO_4_) by average factors of 1.08 and 1.53, respectively. Upon switching to WW, the same ordering of *J*_s_ persisted, with multiplicative increases of 9.29, 5.81, and 6.78 for NaCl, MgSO_4_, and sodium acetate, respectively.

These results indicated that monovalent draw solutes produced the largest reverse salt flux because of their higher ionic mobility and smaller hydration shells compared to those of multivalent ions.^52,53^ Organic draw species showed intermediate behavior (lower mobility than simple monovalent ions but less electrostatic exclusion than multivalent ions), while multivalent ions exhibited the lowest *J*_s_ values due to stronger hydration, larger effective size, and stronger interactions with the membrane charge. The increase in *J*_s_ under WW was attributed to the elevated ionic strength and altered activity coefficients, partial screening of membrane fixed charges (reducing Donnan exclusion), and modified concentration polarization profiles that amplify relative transport differences between draw types.

#### 5.1.3 Specific Reverse Salt Flux *J*_sw_

Figure 4 illustrates the variation in the reverse salt flux (*J*_sw_) across all six experimental combinations, revealing an order-of-magnitude difference in membrane selectivity. Experimental *J*_sw_ values ranged from a minimum of 0.551 mmol L^–1^ to a maximum of 6.930 mmol L^–1^.

The observed trend for *J*_sw_ was marginally declining, almost negligible except when sodium acetate was used as the DS. This steady trend is consistent with the formation of a fouling layer that preferentially impedes reverse solute transport (*J*_s_) relative to water permeation (*J*_w_), thereby producing an apparent improvement in selectivity over time.^25^ In contrast, the NaCl-WW combination exhibited fluctuating behavior (*R* ^2^ = 0.4766), possibly arising from the propagation of independent measurement uncertainties through the ratio *J*_s_/*J*_w_. Since *J*_w_ and *J*_s_ are derived from different instruments, their interval-to-interval measurement errors are statistically independent and combine in quadrature when the ratio is formed, introducing apparent temporal scatter of ±4.1% per measurement point, that the deterministic model does not exhibit by construction. The amplification is configuration-dependent: configurations with larger absolute *J*_s_ values (NaCl/WW) exhibit greater apparent *J*_sw_ scatter because the absolute measurement uncertainty on *J*_s_ scales with its magnitude, while low-*J*_s_ configurations (MgSO_4_) show the least apparent variability.

Switching the feed solution from DI to WW increased *J*_sw_ by a factor ranging from 8.3 to 12.6, a trend attributed to the elevated solute concentration in WW, which simultaneously reduced the net osmotic driving force for *J*_w_ and intensified the concentration gradient for *J*_s_.

The chemical nature of the draw solution significantly influenced selectivity under WW feed conditions. MgSO_4_ yielded the lowest *J*_sw_ in the WW scenario, consistent with the larger hydrated ionic radii and stronger electrostatic interactions of multivalent ions, both of which confer lower salt permeability through the active layer.^48,49,52^ Conversely, NaCl showed higher *J*_sw_ than MgSO_4_ in the WW case, as monovalent ions are typically smaller and diffuse more readily through the membrane. Sodium acetate exhibited the poorest selectivity in the WW case, where despite its molecular size, transport was governed primarily by diffusion and partitioning mechanisms with comparatively weak electrostatic exclusion, facilitating relatively high *J*_s_ values. This apparent inconsistency with sodium acetate’s comparatively moderate absolute *J*_s_ is explained by the denominator of the *J*_sw_ ratio. The disproportionately steep decline in *J*_w_ for sodium acetate under WW feed inflates *J*_sw_ even though its reverse solute flux remains numerically lower than that of NaCl.

The simulated data generally agreed with experimental trends, validating the underlying model. Overall, while feed salinity governed the magnitude of *J*_sw_, the chemical nature of the draw solute (MgSO_4_ > NaCl ≈ Sodium acetate) was the primary factor determining selectivity and the stability of flux decay over the 4-h observation period.

#### 5.1.4 COD Transport, Membrane Selectivity, and Fouling Dynamics

Despite the decline in water flux, the COD solute flux (*J*_COD_) and the specific reverse COD flux (*J*_COD_/*J*_w_ ratio) consistently increased over time for all draw solutions as shown in Figure 5. This behavior indicates that COD transport was primarily governed by diffusion across the transmembrane concentration gradient, rather than by convection-enhanced transport driven by the water flux. This was further reinforced by the progressive build-up of concentration polarization on the draw side, which intensified the COD gradient across the active layer independently of *J*_w_. These flux trends are consistent with the diffusion-dominated regime characterized by *Pe* ≪ 1.

NaCl exhibited the highest *J*_COD_ with a 37.6% relative increase. In contrast, MgSO_4_ showed the lowest *J*_COD_ with the highest COD rejection rate. Sodium acetate demonstrated intermediate values with a relative increase of 44.61% with respect to its own initial flux, reflecting a more pronounced proportional selectivity decline. This inverse relationship between *J*_w_ and *J*_COD_ was attributed to two concurrent mechanisms. Firstly, as water flux declined, the convective transport of COD toward the membrane surface weakened, reducing the capacity to counteract COD back-diffusion and promoting accumulation of an enriched COD layer on the feed side through external concentration polarization. This intensified transmembrane concentration gradient drove greater diffusive COD passage independently of *J*_w_. Secondly, the progressive formation of an initial fouling layer within the 4-hour experimental period gradually reduced the membrane’s apparent rejection capacity, thereby allowing greater COD permeation over time.

The increase in the specific reverse COD flux indicated progressive selectivity decline. MgSO_4_ maintained the lowest ratio with only 27.0% increase, while NaCl and sodium acetate experienced 59.57% and 55.9% increases, respectively, suggesting that the physicochemical properties of the MgSO_4_ draw solute provided superior selectivity preservation.

Water recovery for NaCl, MgSO_4_ and sodium acetate was 19%, 9.4%, and 11.3%, respectively (Figure 6), reflecting differences in osmotic driving force and initial fouling characteristics. The volume concentration factor (CF_v_) was 1.23, 1.10, and 1.13 respectively, with NaCl achieving the greatest feed concentration.

**Figure 6:**
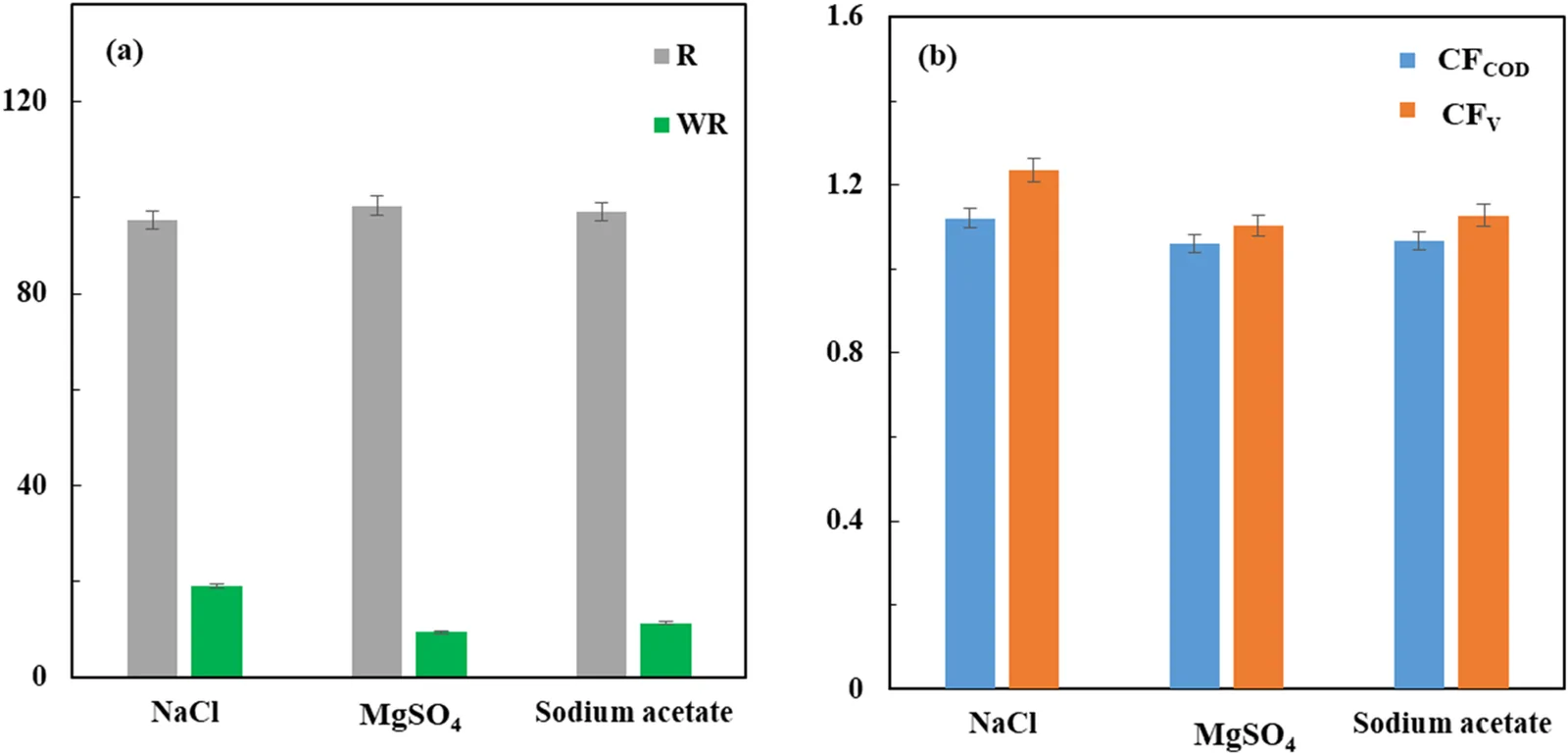
(a) Final COD rejection rate (*R*) and water recovery (WR), and (b) final volume concentration factor (CF_v_), COD concentration factor (CF_COD_) for three draw solutions.

The COD concentration factor (CF_COD_) values (Figure 6) of 1.12, 1.06, and 1.07 approached their corresponding CF_v_ values, indicating that COD concentration scaled nearly proportionally with water removal, consistent with near-ideal rejection behavior. COD rejection rates for NaCl, MgSO_4_ and sodium acetate were 95.40%, 98.30%, and 97.04%. MgSO_4_ exhibited superior rejection, likely due to larger hydrated ionic radii and enhanced electrostatic interactions with the membrane, while sodium acetate’s rejection was more plausibly influenced by size exclusion and hydrophobic partitioning.^54^

At the end of the experiment, the accumulated fouling mass was 0.137, 0.078, and 0.153 g m^–2^ for NaCl, MgSO_4_, and sodium acetate respectively. MgSO_4_ exhibited 43% lower fouling than NaCl and 49% lower than sodium acetate over the 4-h experimental period, demonstrating superior resistance to initial foulant deposition under the tested conditions. The higher initial fouling mass under sodium acetate within the 4-h window is consistent with enhanced external concentration polarization. An additional contribution from the hypothesized tendency of the organic draw solute to promote a more cohesive foulant layer through hydrophobic interactions and adsorption onto the active layer cannot be excluded. However, direct confirmation would require post-experiment surface analysis.

The flux decline per gram of accumulated foulants over the 4-h experimental period was 3.82, 1.31, and 1.06 L g^–1^ h^–1^ for NaCl, MgSO_4_, and sodium acetate respectively. NaCl’s higher flux-decline-per-gram value is consistent with a more hydraulically resistive foulant layer, possibly reflecting denser cake packing. In contrast, the lower values for MgSO_4_ and sodium acetate are consistent with more open foulant structures, possibly arising from differences in foulant composition, electrostatic repulsion, or packing density.^55^ This indicates that the severe water-flux decline observed for sodium acetate (Section 5.1.2) is not primarily attributable to fouling-layer hydraulic resistance, despite its highest total fouling mass. Rather, it is more consistent with the viscosity and hydrophobic-interaction effects, with fouling acting as a secondary, lower-impact contributor for this draw solute. As no direct surface characterization of the fouled coupons was performed, the cake-structure interpretations above carry the same evidentiary caveat stated in Section 2.2.

### 5.2 Validation of the Prediction Tool

#### 5.2.1 Validation of the Prediction Tool for *J*_w_

The predictive model demonstrated high fidelity in reproducing experimental water-flux trajectories across all DS/FS combinations, as presented in Table 4. Accounting for the combined experimental measurement uncertainty of ±1% on *J*_w_, the observed RRMSE values of below 1.9% indicate that the true model error above the experimental noise floor does not exceed 1.6%, confirming that the model predictions are statistically indistinguishable from the experimental data within measurement precision.

**Table 4:** Regression Statistics for *J*_w_ Model Validation across All DS/FS Combinations

*J*_w_ = *f*(*J*_w,exp_)	*a*	*R* ^2^	RMSE (L m^–2^ h^–1^)	RRMSE (%)	MRE (%)
Sodium acetate/DI	1.005	0.911	0.030	0.74	2.10
Sodium acetate/WW	1.01	0.635	0.040	1.87	3.29
NaCl/DI	1.001	0.971	0.035	0.89	2.48
NaCl/WW	0.976	0.933	0.056	1.56	4.54
MgSO_4_/DI	0.987	0.593	0.030	1.17	3.06
MgSO_4_/WW	1.025	0.605	0.026	1.44	4.83

Regression analysis constrained through the origin yielded slopes tightly clustered between 0.976 and 1.025, indicating less than 3% proportional deviation from experimental values. Specifically, the model for NaCl exhibited minimal bias across both feed solutions, achieving the best agreement for NaCl/DI (*R* ^2^ = 0.971, *a* = 1.001, RRMSE = 0.89%, maximum relative error (MRE) = 2.5%). Sodium acetate maintained magnitude accuracy (RRMSE < 1.9%, *a* ≈ 1.00). However, its *R* ^2^ decreased from 0.91 to 0.64 with wastewater owing to pronounced non-systematic scatter, indicating that the model continued to reproduce the mean flux trajectory accurately, but did not capture point-to-point variability. MgSO_4_ combination also showed reduced R^2^ values (≈0.6) irrespective of the feed, attributed to model limitations such as omitting the Donnan effect and dielectric exclusion.

#### 5.2.2 Validation of the Prediction Tool for *J*_s_

As summarized in Table 5, the model for *J*_s_ generally reproduced measured values with acceptable fidelity exhibiting *R* ^2^ values ranging from 0.71 to 0.95 and regression slopes between 0.84 and 1.02, indicating less than 16% proportional deviation in the worst case. Given the combined experimental uncertainty of ±3.5% on *J*_s_ arising from inter-run reproducibility and conductivity probe accuracy, the observed RRMSE values of 1.7–5.9% indicate that approximately half of the reported model-data discrepancy is attributable to irreducible measurement noise rather than model structural error. The true model error above the experimental noise floor does not exceed 4.7% across all configurations, confirming that the model reproduces the salt transport dynamics within the bounds imposed by the measurement system.

**Table 5:** Regression Statistics for *J*_s_ Model Validation across all DS/FS Combinations

*J*_s_ = *f*(*J*_s,exp_)	*a*	*R* ^2^	RMSE (mol m^–2^ h^–1^)	RRMSE (%)	MRE (%)
Sodium acetate/DI	0.853	0.953	8.0 × 10^–5^	3.60	18.49
Sodium acetate/WW	1.014	0.714	8.84 × 10^–4^	5.90	10.24
NaCl/DI	0.951	0.916	4.09 × 10^–5^	1.74	9.42
NaCl/WW	0.984	0.856	7.82 × 10^–4^	3.51	5.78
MgSO_4_/DI	0.837	0.821	8.65 × 10^–5^	5.52	24.52
MgSO_4_/WW	0.969	0.851	2.21 × 10^–4^	2.43	7.74

Optimal agreement was achieved for NaCl (*R* ^2^ ≥ 0.86, RRMSE ≤ 3.5%) and maximum relative errors remained below 9.5% across both feeds, physically consistent with the monovalent, fast-diffusing character of Na^+^ and Cl^–^ ions. Sodium acetate and MgSO_4_, particularly with DI feed, introduced additional scatter and systematic negative biases, with scaling factors dropping to 0.85 and 0.84 for sodium acetate/DI and MgSO_4_/DI, respectively. Despite high R^2^ values (0.82–0.88) in these cases, maximum relative errors surged to 24.5%. This indicated that the model correctly reproduced the shape of *J*_s_ but under-predicted its magnitude.

Therefore, the model was deemed quantitatively reliable for NaCl across both feed conditions and for sodium acetate/WW and MgSO_4_/WW. Its quantitative reliability for sodium acetate/DI and MgSO_4_/DI is compromised by the systematic under-prediction attributed to unmodelled electrostatic screening and specific ion interactions.

#### 5.2.3 Validation of the Prediction Tool for COD

The simulated COD_D_ and COD_F_ concentrations are presented alongside experimental values in Figure 7. Table 6 reports the corresponding regression statistics for COD model validation after error minimization, separately for the feed and draw solutions. Both variables showed high coefficients of determination (*R* ^2^ ≈ 0.975–0.996). However, the relative error metrics revealed a marked disparity, as COD-F was reproduced with high accuracy (RRMSE ≈ 0.35–0.53%, MAE ≈ 0.3–0.4%), while COD-D exhibited substantially larger relative errors (RRMSE ≈ 8–9%, MAE ≈ 6.8–7.2%).

**Figure 7:**
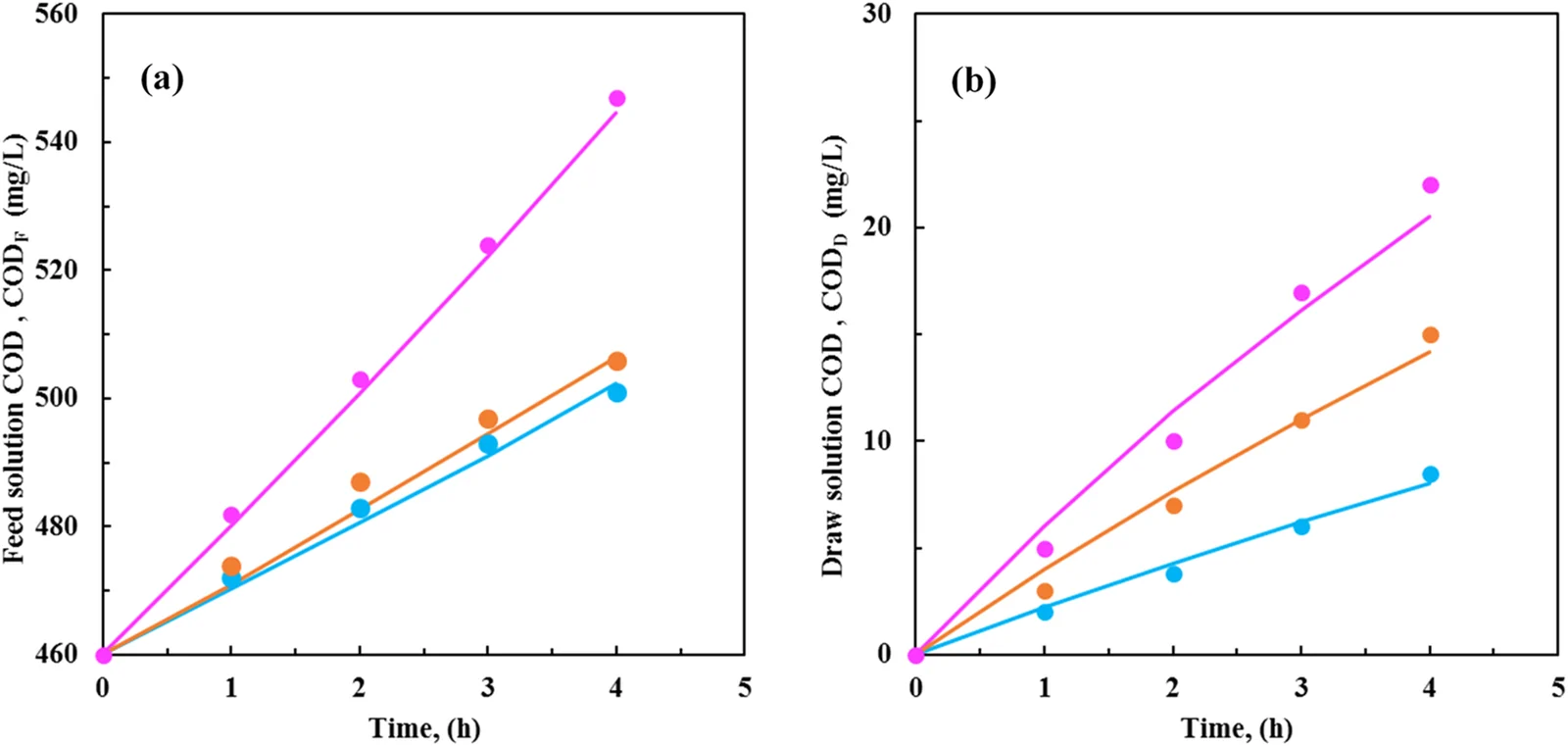
Experimental vs predicted COD for both feed (a) and draw (b) solutions.

**Table 6:** COD Validation Regression Statistics

COD = *f*(COD_exp_)		*a*	*R* ^2^	RRMSE (%)	MAE (%)	MBE (%)
NaCl	COD_D_	0.969	0.980	8.509	7.194	2.128
	COD_F_	0.997	0.999	0.376	0.333	-0.333
MgSO_4_	COD_D_	0.997	0.986	8.157	6.847	2.236
	COD_F_	0.9981	0.991	0.349	0.306	-0.190
Sodium acetate	COD_D_	0.9906	0.984	9.013	7.001	2.563
	COD_F_	0.9963	0.990	0.529	0.419	-0.375

Since all metrics were normalized, this discrepancy could not be attributed to the difference in magnitude between the two variables. Instead, it reflected the inherent asymmetry of the parameter identification system. COD_F_ functioned as the dominant directly constrained state variable, whereas COD_D_ behaved as a more sensitive dependent variable, strongly affected by small inaccuracies in transport terms. The opposite signs of the mean bias error (negative for COD_F_ and positive for COD_D_) further indicated an error redistribution imposed by the mass balance constraint. In practical terms, this uncertainty in COD_D_ prediction carries a bounded and manageable consequence for process design. The absolute COD_D_ uncertainty corresponding to 0.9 mg L^–1^ remains several orders of magnitude below the feed-side concentrations.

The disparity between COD_F_ and COD_D_ prediction accuracy is further explained by the difference in their respective experimental measurement uncertainties. The combined uncertainty on COD_F_ measurements is ±1.5%, arising primarily from the colorimetric analytical precision. The RRMSE of 0.35–0.53% for COD_F_ therefore falls entirely below this uncertainty floor, confirming that the model prediction for feed-side COD is statistically indistinguishable from a perfect prediction given the resolution of the measurement system. For COD_D_, the combined experimental uncertainty is ±5.5%, elevated relative to COD_F_ owing to the amplification of analytical uncertainty at the low absolute concentrations present on the draw side during the early experimental period. The observed RRMSE of 8–9.1% for COD_D_ therefore contains an irreducible noise contribution of 5.5%. The true model structural error above this noise floor does not exceed 7.1% across all draw solution configurations.

The calibrated coefficients for the three draw solutions are presented in Table 7. P_COD_ values ranged from 0.185 L m^–2^ h^–1^ to 0.506 L m^–2^ h^–1^, representing approximately 3-fold variation across draw solutions, all within the admissible range defined in Section 3.2 (0.005–0.5 L m^–2^ h^–1^). NaCl exhibited the highest *P*_COD_ and marginally exceeding the literature upper bound by 1.2%. This high value is attributed to the combination of high water flux and strong external CP on the feed side, both of which enhance the effective transmembrane concentration gradient for dissolved organics, consistent with the simultaneously elevated *K*_foul_ value for this configuration.

**Table 7:** Predicted *P*_COD_, *K*_foul_ and *P*_COD_/*K*_foul_ for the Different Draw Solutions

parameter	NaCl	MgSO_4_	sodium acetate
*P*_COD_ (L m^–2^ h^–1^)	0.506	0.185	0.306
*K*_foul_ (L m^–2^ h^–1^)	0.0824	0.0405	0.079
*P*_COD_/K_foul_ (−)	6.14	4.58	3.87

MgSO_4_ exhibited the lowest *P*_COD_, consistent with stronger electrostatic interactions and lower water flux, providing superior COD rejection. Sodium acetate occupies a unique position among the three draw solutions because the draw solute itself carries an intrinsic COD. To isolate the feed-origin organic permeation flux from the draw-solute contribution, a blank correction was applied at each time point using the conductivity-derived draw-solute concentration (Section 2.3). After correction, sodium acetate exhibited intermediate *P*_COD_ values (Table 7), confirming that its feed-origin organic transport lies between NaCl (highest) and MgSO_4_ (lowest).

The calibrated *K*_foul_ values ranged from 0.0405 L m^–2^ h^–1^ to 0.0824 L m^–2^ h^–1^, representing a 2-fold variation, all within the practical FO wastewater range (0.01–0.5L m^–2^ h^–1^). NaCl showed the highest *K*_foul_ associated with high convective foulant transport and weak electrostatic repulsion. MgSO_4_ exhibited the lowest *K*_foul_, through strong electrostatic repulsion and dense cake layer formation. Sodium acetate showed *K*_foul_ comparable to NaCl, with organic draw solute promoting hydrophobic foulant interactions. An important consideration is the reverse flux of acetate from draw to feed: any acetate permeating back into the feed solution increases the measured feed-side COD over time, which would cause the model to overestimate COD concentration enhancement and underestimate leakage for this configuration. This effect is implicitly captured in the calibrated *K*_foul_ term but cannot be independently resolved without speciated organic analysis.

To quantify pathway contributions, the time-integrated COD mass transferred via leakage and deposited via fouling was computed from the calibrated model over the 4-hour period. Leakage accounted for 82–89% of total COD removal across the three configurations, with fouling deposition contributing the remaining 11–18%, confirming a leakage-dominated regime consistent with the *P*_COD_/*K*_foul_ ratios in Table 7.

The dimensionless ratio *P*_COD_/*K*_foul_ also provided a quantitative measure of the selectivity trade-off between COD leakage and foulant deposition, with higher ratios indicating preferential transport of COD relative to foulant accumulation. NaCl exhibited the highest selectivity ratio, indicating that within the 4-hour experimental period COD transport was 6.1 times higher than the initial fouling deposition rate, suggesting a leakage-dominated regime necessitating frequent cleaning and limiting long-term operational sustainability. MgSO_4_ exhibited an intermediate selectivity ratio, indicating a balanced system where both transport and fouling were minimized through electrostatic mechanisms, making it the preferable candidate for applications prioritizing selectivity and fouling resistance. Sodium acetate exhibited the lowest selectivity ratio, indicating a fouling-dominated initial deposition regime within the 4-h window that was less suitable for sustained operation despite moderate COD leakage.

### 5.3 Effect of Concentration Polarization

Figure 8 illustrates the contributions of ECP-DS, ICP, effective osmotic pressure, and ECP-FS to total osmotic driving force attenuation across all DS/FS combinations. It reveals that internal concentration polarization (ICP) was the primary factor limiting the effective driving force, consistently accounting for the largest osmotic pressure drop.

**Figure 8:**
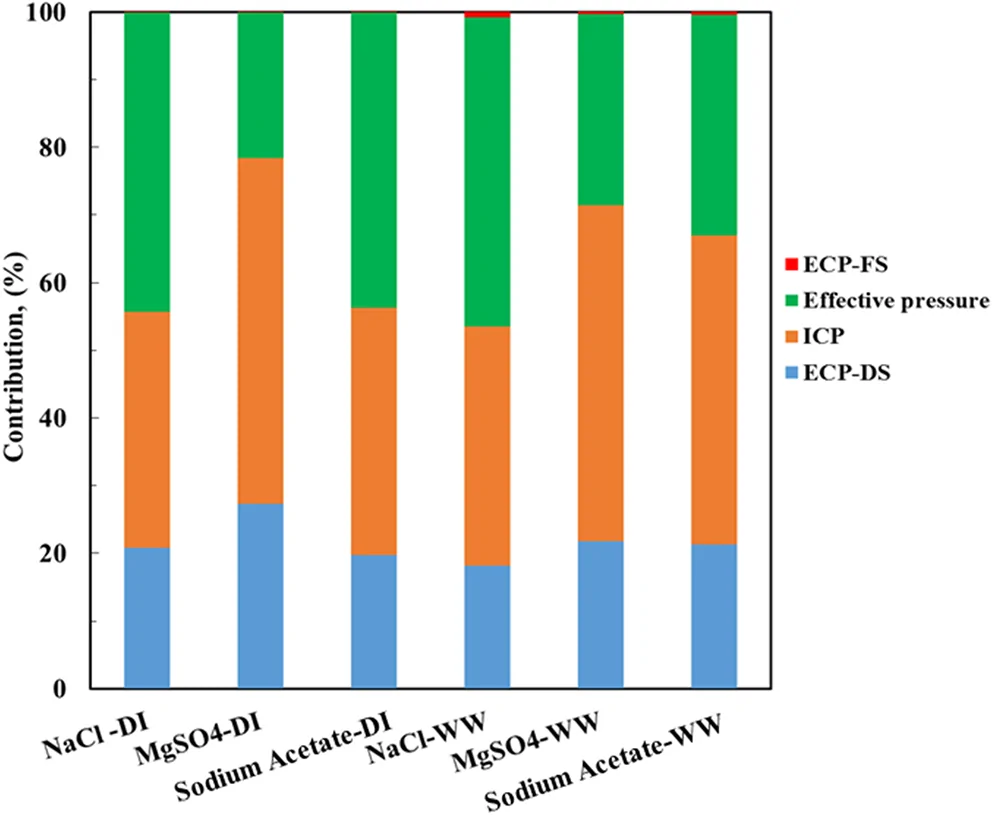
Percentage contributions of ECP-DS, ICP, effective osmotic pressure, and ECP-FS to total osmotic driving force attenuation across all DS/FS combinations.

For MgSO_4_ draw solution in particular, ICP exceeded 50% due to the lower diffusivity and larger hydrated radii of multivalent ions (Mg^2+^ and SO_4_^2–^), which severely constrained their mass transfer within the membrane’s porous support layer.^49^ It is important to distinguish here between the steady-state magnitude of CP-induced driving-force loss, and the sensitivity of that loss to a change in feed composition. The first reflects the equilibrium transport resistance imposed by a given draw solute, while the second reflects how much additional perturbation the system can absorb before the driving force is further degraded. These two properties are not necessarily correlated.

Although MgSO_4_ exhibited the highest ICP magnitude, its temporal flux decay remained the most stable (*k* and RCL nearly unchanged between DI and WW runs). This apparent discrepancy reflects the fact that ICP layer is already near-saturated at the outset for MgSO_4_. Thus, incremental perturbations from WW solutes produce proportionally smaller relative changes in the effective driving force. By contrast, NaCl operates with lower baseline ICP, retaining greater headroom for WW-induced amplification of concentration gradients within the support, producing steeper and more variable flux decline kinetics.

External Concentration Polarization on the Draw Solution side (ECP-DS) also contributed notably, accounting for 18% and 28% of total osmotic pressure attenuation, as solute accumulated at the active layer surface due to limited back diffusion. NaCl draw solution, conversely, exhibited the highest effective osmotic pressure (up to 45.64% under WW conditions) reflecting its smaller hydrated radius and higher diffusivity, which mitigated both ICP and ECP-DS more effectively than multivalent or organic species. Sodium acetate, characterized by a larger molecular size and a lower diffusivity than monovalent ions, also demonstrated significant ICP (36.55–45.75%), leading to moderate effective pressures.

The transition from DI to WW as the feed solution introduced distinct effects on CP. The presence of dissolved solutes in WW increased the external concentration polarization on the feed solution side (ECP-FS), from negligible levels (0.02% under DI) to up to 0.79% under WW, as feed-side solutes accumulated at the membrane interface and generated an opposing osmotic pressure.

Notably, for ionic draw solutions, the simulation predicted an increase in effective pressure when using WW as a feed solution, ranging from 44.34% to 45.64% and from 21.53% to 28.28% for NaCl and MgSO_4_ draw solutions, respectively. This increase in the effective pressure fraction was not due to an absolute increase in the driving force, but rather a consequence of the overall reduction in water flux when operating with WW. A lower *J*_w_ inherently led to less severe concentration polarization effects, both dilutive (ECP-DS) and concentrative (ECP-FS, ICP), thereby causing the interfacial concentrations to move closer to their respective bulk values. Consequently, the attenuation of the osmotic driving force due to CP was reduced in magnitude, resulting in a higher fraction of the bulk osmotic pressure being effectively utilized. However, despite this relative increase in the effective pressure fraction, it did not translate into higher *J*_w_ because WW simultaneously introduced additional hydraulic resistance through incipient fouling layer formation and enhanced ECP-FS, which collectively outweighed any apparent driving force gain.

In contrast, the sodium acetate draw solution did not benefit from this ECP-DS mitigation mechanism. Consequently, the increased ECP-FS from WW directly reduced its effective osmotic pressure from 43.75% to 32.49%, the largest absolute reduction among all draw solutes. This is consistent with the greatest *J*_w_ decline under WW conditions.

The dominance of ICP in the CP budget observed here for all three draw solutions is consistent with the broader osmotically assisted reverse osmosis (OARO) and FO transport literature, in which ICP is identified as the primary flux-limiting mechanism in asymmetric membranes with S > 300 μm operating under high osmotic pressure driving forces.^56^ The differential ICP magnitude across draw solutions, with the divalent draw solution exhibiting the largest ICP fraction, reflects the well-documented inverse relationship between ion diffusivity and ICP severity in confined pore geometries, a relationship that has recently been quantified under multi-electrolyte conditions in both FO and OARO contexts.^47,57^ The observation that ECP-FS increased substantially on switching from DI to WW feed is also consistent with literature showing real wastewater feeds intensify feed-side concentration polarization, whether through fouling-enhanced CP or reduced boundary-layer mass transfer.^58^

### 5.4 Economic Performance, Uncertainty Quantification, and Sensitivity Analysis

#### 5.4.1 Economic Performance

Figure 9 illustrates the Levelized Cost of Water (LCOW) for FO when employing wastewater as the feed solution. The techno-economic analysis identified NaCl as the most economically viable draw solution, achieving the lowest levelized cost of water of USD 1.04 m^–3^, approximately 40% below both MgSO_4_ (USD 1.75 m^–3^) and sodium acetate (USD 1.74 m^–3^), which performed comparably despite differing cost structures.

**Figure 9:**
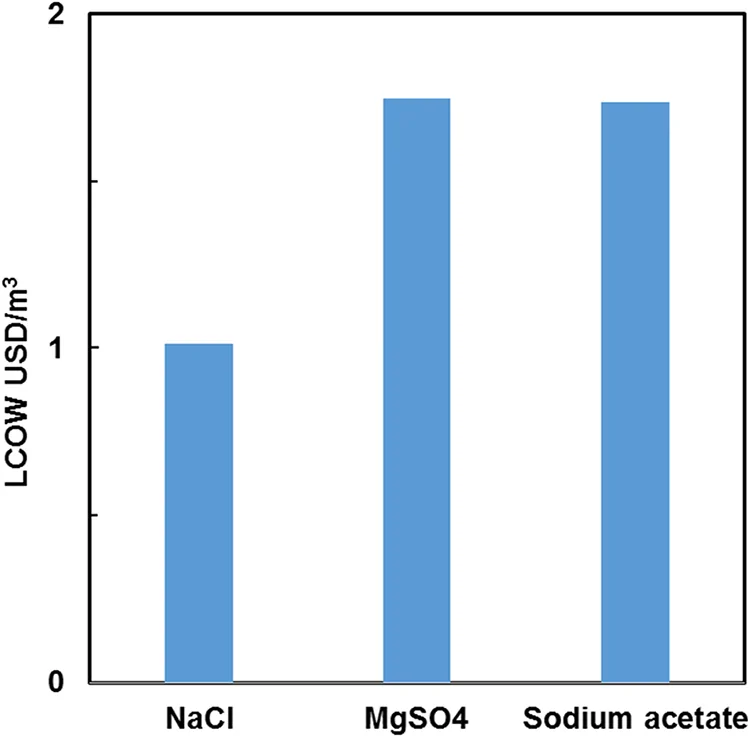
LCOW values for different combinations.

The cost distribution analysis, presented in Figure 10, revealed a structurally distinct economic profile for each draw solution, reflecting the coupled influence of transmembrane water flux, draw solution osmotic pressure, and draw solute unit market price on the overall process economics. Among the draw solutions evaluated, NaCl yields the lowest levelized cost of water (LCOW), supported by a well-balanced cost structure in which no single expenditure category constitutes a dominant vulnerability. This value falls within the range reported for FO wastewater treatment systems in recent literature (USD 0.37–1.47 m^–3^).^18–20^ OPEX_RM_ leads at 32.0%, followed by CAPEX_Memb_ capital (16.3%), OPEX_clean_ (14.1%), and OPEX_energy_ (14.0%), while draw solute replenishment, OPEX_solute_, remains negligible at 3.0%, reflecting sodium chloride’s commodity pricing. The MgSO_4_ system is co-dominated by OPEX_RM_ and CAPEX_Memb_, each running approximately twice and 93% above their NaCl equivalents, respectively, a direct consequence of the lower water flux characteristic of divalent solutes. OPEX_solute_, though moderate in share (8.2%), is five times the NaCl absolute value. Sodium acetate arrives at a near-identical total cost to MgSO_4_ but through an entirely different cost profile: OPEX_solute_ alone accounts for 19.7%, eleven times the NaCl reference, driven by sodium acetate’s elevated unit procurement cost.

**Figure 10:**
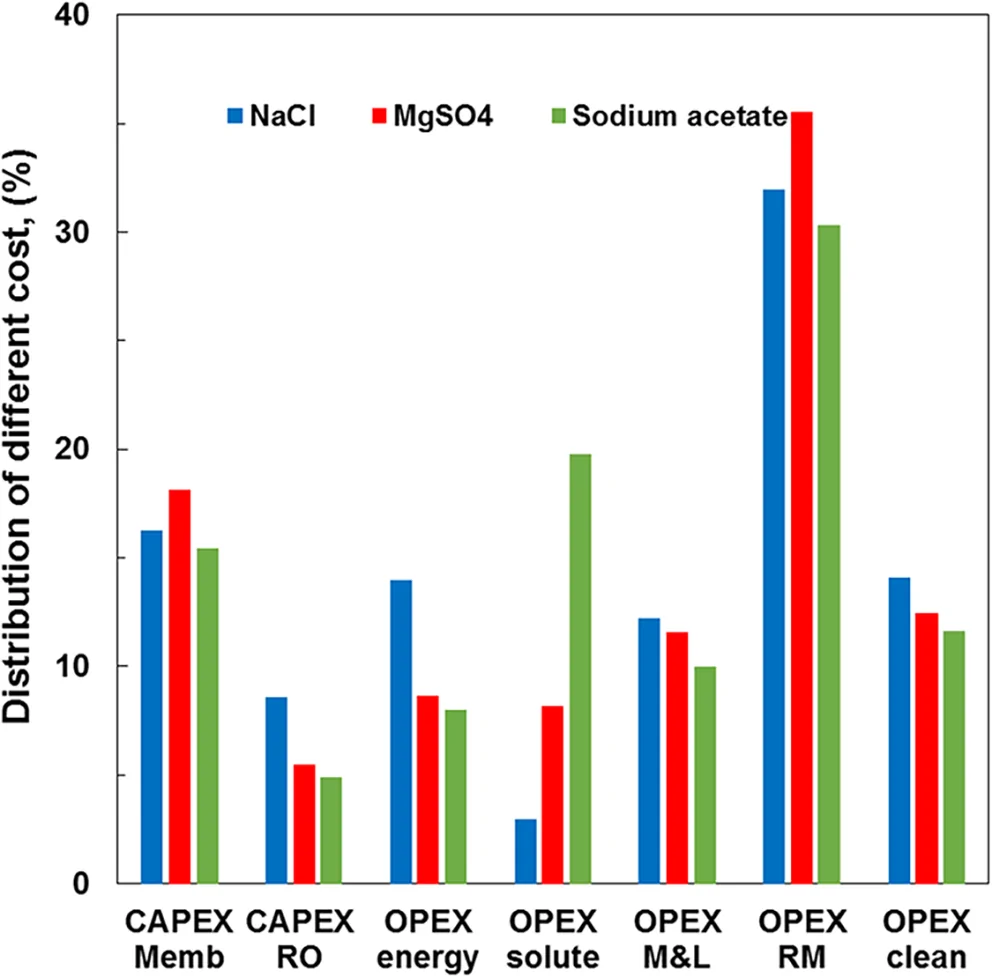
Distribution of different costs.

NaCl’s advantage reflects a structural rather than marginal difference in cost performance. It indicates uniformly lower absolute expenditure across all categories, meaning the gap cannot be closed by optimizing any single driver; it is an inherent consequence of the physicochemical limitations of divalent and organic draw agents. Between MgSO_4_ and sodium acetate, near-equivalent total LCOWs of USD 1.75 m^–3^ and USD 1.74 m^–3^ mask a fundamental redistribution of cost burden: flux-driven capital and OPEX_RM_ penalties for the former, OPEX_solute_ for the latter. The apparent OPEX_energy_ intensity advantage inferred from percentage shares (14.0%, 8.65%, and 8.02% for NaCl, MgSO_4_, and sodium acetate) is a consequence of differences in total LCOW denominators rather than differences in absolute energy expenditure.

#### 5.4.2 Uncertainty Quantification and Sensitivity Analysis

The resulting LCOW distributions, presented in Figure 11, yielded mean estimates of USD 1.04 m^–3^ (95% CI: 0.97–1.12), USD 1.75 m^–3^ (95% CI: 1.63–1.93), and USD 1.74 m^–3^ (95% CI: 1.64–1.95) for NaCl, MgSO_4_, and sodium acetate, respectively. NaCl achieved a lower LCOW in all Monte Carlo realizations with completely non-overlapping intervals compared to MgSO_4_ and sodium acetate.

**Figure 11:**
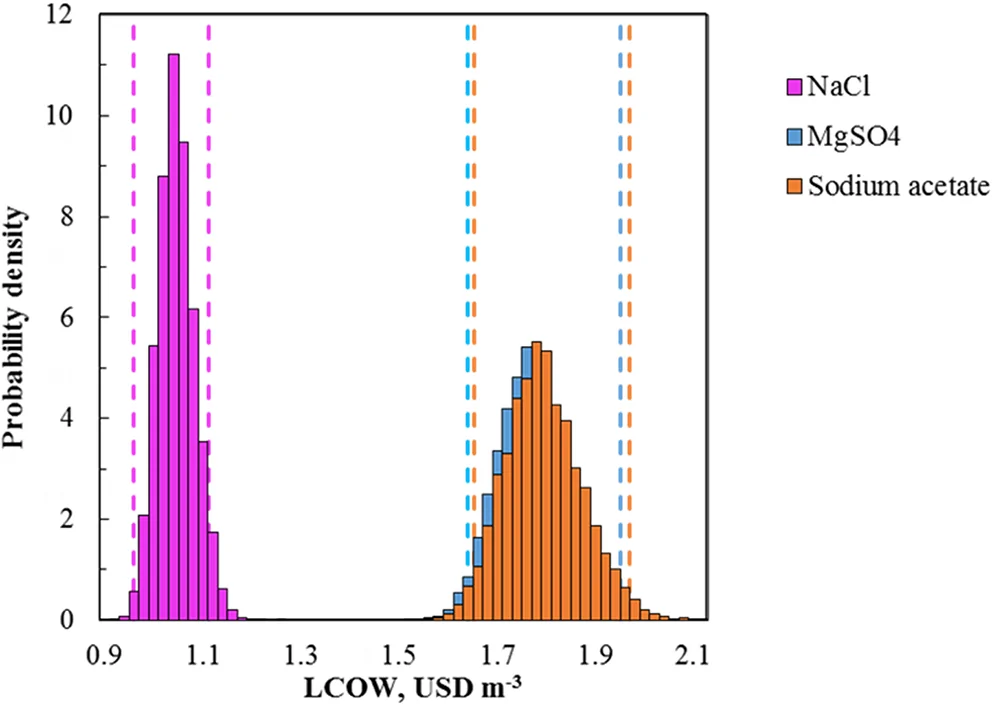
Probability density distributions of LCOW for the three draw solutions. Dashed vertical lines denote the 2.5th and 97.5th percentiles of each.

This indicates an estimated 100% probability that NaCl yielded the lowest LCOW among all compared designs. It also confirmed that the economic advantage of NaCl is statistically robust with respect to experimental flux uncertainty and could not be attributed to measurement imprecision. MgSO_4_ and sodium acetate, by contrast, yielded substantially overlapping confidence intervals, indicating that their LCOW values are not statistically distinguishable at the 95% level. Their comparative evaluation must therefore rest on selectivity, fouling resistance, and draw solute procurement cost rather than on economic grounds alone.

All three distributions exhibited the right-skewed profile expected from the nonlinear 1/*J*_w_ dependence of membrane area, whereby a downward perturbation in flux inflates LCOW by a disproportionately larger margin than an equivalent upward perturbation reduces it. Notably, the absolute width of the 95% confidence interval for MgSO_4_ and sodium acetate (approximately USD 0.26–0.27 m^–3^) was nearly twice that of NaCl (approximately USD 0.15 m^–3^), a direct consequence of the nonlinear amplification of flux uncertainty at lower mean flux values where membrane area is substantially larger. The relative uncertainty was consistent across all three configurations at approximately 3.7%, confirming that the broader absolute confidence intervals arise from their higher baseline cost rather than from any difference in experimental measurement precision.

Sensitivity analysis across membrane unit cost, water flux, discount rate, and electricity price, presented in Figure 12, confirms that the systems differ not only in baseline cost but in their exposure to uncertainty. Water flux is the most nonlinearly sensitive parameter: at 1 L m^–2^ h^–1^, LCOW reaches USD 2.483 m^–3^, USD 2.753 m^–3^, and USD 3.184 m^–3^ for NaCl, MgSO_4_, and sodium acetate, respectively, declining sharply toward a plateau at 7-9 L m^–2^ h^–1^ as fixed costs dominate. Sodium acetate incurs the steepest low-flux penalty because its high solute replenishment cost inflates every fixed expenditure on a per-volume basis, making sustained high-flux operation the highest single-impact technical objective across all systems.

**Figure 12:**
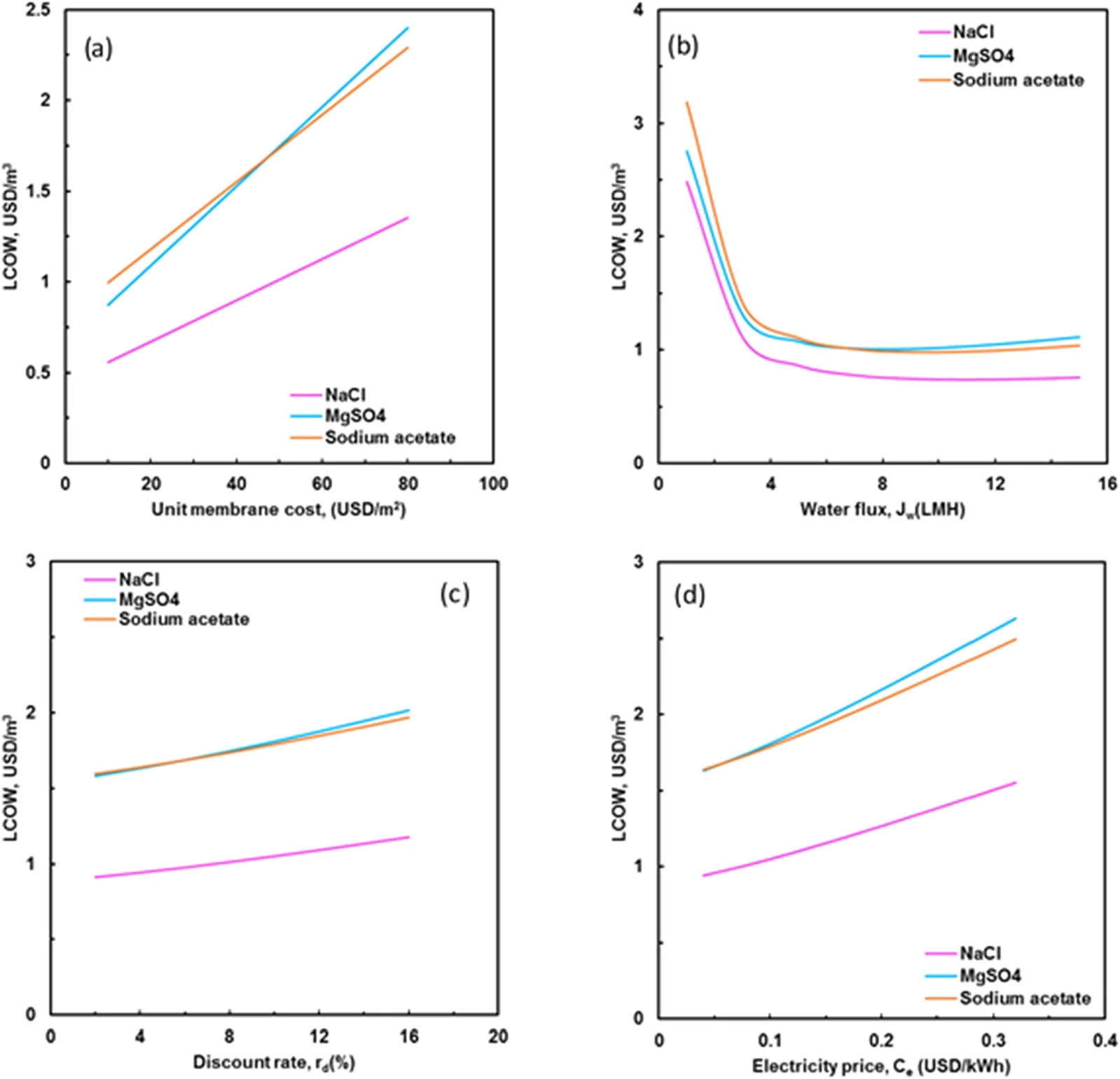
LCOW sensitivity to membrane cost (a), water flux (b), discount rate (c), and electricity price (d).

Membrane unit cost exerts a quasi-linear effect, with LCOW varying by USD 0.796 m^–3^, USD 1.528 m^–3^, and USD 1.296 m^–3^ over USD 10–80 m^–2^. The NaCl advantage persists even at minimum membrane prices, confirming it is not a capital cost artifact. Discount rate sensitivity produces gradients of USD 0.018 m^–3^ versus USD 0.029 m^–3^ per percentage point for NaCl relative to the other systems. At 20% discount rate, MgSO_4_ and sodium acetate incur increases of USD 0.418 m^–3^, more than double NaCl ’s USD 0.256 m^–3^, disproportionately penalizing these systems under high-cost financing. Electricity prices variation over USD 0.04-0.40 kWh^–1^ produces swings of USD 0.807 m^–3^, USD 1.321 m^–3^, and USD 1.132 m^–3^, with MgSO_4_ and sodium acetate exhibiting wider ranges despite similar baseline energy costs, an amplification effect of their larger total cost bases.

Water flux and membrane cost are the primary technical levers for LCOW reduction; discount rate and electricity price represent the dominant external risk factors. Across all four dimensions, MgSO_4_ and sodium acetate exhibit greater absolute exposure than NaCl, reinforcing the structural resilience of monovalent draw solutions under techno-economic uncertainty.

The resulting LCOW range attributable to cleaning frequency uncertainty, due to cleaning trigger factors, was USD± 0.07 m^–3^ for NaCl, USD ± 0.11 m^–3^ for MgSO_4_, and USD ±0.09 m^–3^ for sodium acetate, confirming that this source of uncertainty does not alter the comparative economic ranking of the three draw solutions. The baseline scenario is used for all reported deterministic LCOW values.

## 6 Overall Performance Trade-Off and Draw Solution Selection

The comparative analysis revealed a multidimensional trade-off among the three draw solutions that extended beyond hydraulics to encompass membrane selectivity, fouling tendencies, and economic viability. Thus, the optimal selection is inherently application-specific. The decision framework maps out actions across three distincts scenarios: Cost-constrained high-throughput water production, Effluent quality-constrained or high-value resource recovery applications, and Applications with strict draw-solute environmental or regulatory constraints.

NaCl emerged as the most economically favorable option, generating the highest values of permeate flux, water recovery, volumetric concentrate ratio, and COD concentration factor, thereby reducing the total membrane area required and lowering the LCOW. These features made NaCl the most suitable draw solution for maximizing process output and minimizing costs. However, these performance advantages were obtained at the expense of high reverse salt flux and greater initial foulant accumulation within the 4-hour experimental window under wastewater conditions. COD transfer to the draw solution, the primary operational challenge, is most appropriately managed through downstream draw-solution polishing or by accepting higher organic load on the RO regeneration unit where effluent quality requirements are moderate.

MgSO_4_, by contrast, provided the best COD removal efficiency, the lowest initial fouling accumulation within the 4-hour experimental period, and the most stable flux decay kinetics under the tested conditions. Although MgSO_4_ required more membrane area, leading to a higher LCOW, this draw solution was identified as the most suitable candidate for applications in which selectivity, fouling resistance, and operational stability are prioritized over cost minimization, such as high-value resource recovery or sensitive effluent treatment scenarios.

Sodium acetate was positioned hydraulically midway between MgSO_4_ and NaCl but was at a disadvantage structurally driven by its expensive draw-solute replenishment, which rendered it uncompetitive. Nonetheless, sodium acetate presents three deployment scenarios where it may be preferred over NaCl despite its higher LCOW:

(i) Jurisdictions where NaCl discharge restrictions apply due to receiving-water salinity limits, since acetate is fully biodegradable and environmentally benign;
(ii) FO–biological treatment hybrids where low-level reverse acetate flux into the feed stream serves as a supplementary carbon source for downstream biological polishing, turning a process inefficiency into a functional advantage;
(iii) Applications requiring food-grade or pharmaceutical-grade product water compatibility, where inorganic salt contamination is unacceptable.

In all other scenarios, the structural LCOW penalty, driven by acetate’s unit market price, renders it uncompetitive without a quantifiable premium attributable to its environmental or process co-benefit.

Overall, these findings confirmed that draw solution selection for FO cannot be reduced to a single performance parameter. The best choice depends on the application requirements and the relative weighting of cost, selectivity, fouling resistance, and environmental compliance.

## 7 Conclusion

This study presented a combined experimental, modeling, and techno-economic investigation of FO for wastewater treatment, providing a quantitative characterization of water, solute, and organic matter transport across three draw solutions and two feed conditions.

The experimental campaign confirmed that the transition from deionized water to real wastewater feed induced flux declines ranging from 8.14% to 46.19% over the 4-h experimental period, attributable to the combined effect of organic fouling and increased feed-side osmotic resistance, with NaCl consistently exhibiting the highest water flux. The MATLAB finite-difference model, incorporating a bisection solver for water flux estimation and empirical correlations for solution properties, accurately reproduced the experimental *J*_w_ and *J*_s_ profiles and the full-transmembrane concentration profiles, validating the numerical framework as a reliable predictive tool for FO system simulation under complex feed conditions.

The phenomenological COD sub-model introduced in this work allowed the decoupling of organic matter dynamics into three physically distinct contributions: feed-side COD concentration enhancement, transmembrane leakage governed by *P*_COD_, and irreversible fouling accumulation governed by *K*_foul_. The calibrated parameters revealed that transmembrane leakage was the dominant pathway for COD loss from the feed liquid phase under the tested conditions, accounting for 82–89% of total COD mass transferred out of the feed reservoir, while irreversible fouling deposition contributed 11–18%. It is important to note that transmembrane leakage does not represent effective treatment: COD that permeates the membrane transfers to the draw solution rather than being removed from the system. This contamination of the draw solution has two process implications. Firstly, it reduces the apparent purity of the regenerated draw solution and may impose an additional organic load on the RO regeneration unit. Secondly, true irreversible COD removal from the liquid phase is governed exclusively by the fouling deposition term, which was substantially lower than the leakage term across all draw solutions. The dimensionless *P*_COD_/*K*_foul_ ratio introduced here provides a practical single-parameter screening metric for draw solution selectivity. MgSO_4_ exhibited the lowest *K*_foul_ of 0.04 L m^–2^ h^–1^ and the most balanced *P*_COD_/*K*_foul_ ratio, indicating that both leakage-driven draw-solution contamination and irreversible fouling accumulation were minimized relative to the other draw solutes.

The techno-economic analysis identified NaCl as the most economically viable draw solution for FO wastewater treatment, achieving a levelized cost of water of USD 1.04 m^–3^, 40% lower than the worst-performing configuration. Sensitivity analysis confirmed that transmembrane water flux is the primary technical lever governing LCOW, while discount rate and electricity price represent the dominant external risk factors. The modeling framework developed here constitutes a transferable, experimentally grounded tool for draw solution screening and FO system design. The Monte Carlo analysis demonstrated that the economic ranking is statistically robust across the full joint uncertainty space of the experimental flux measurements.

Several limitations should be acknowledged. WW characterization was limited to COD and osmotic pressure, which precludes assessment of how specific feed matrix components contribute to the observed fouling patterns. Foulant accumulation was quantified through COD mass balance alone, capturing only the organic fraction of total foulant accumulation. The 4-h experimental window characterizes the initial-to-intermediate fouling regime, during which first-order fouling kinetics is a valid modeling assumption, but does not capture biofouling dynamics, cake compaction, or cleaning-cycle recovery, all of which emerge under extended continuous operation. Therefore, the comparative fouling rankings reported should be interpreted as initial-regime indicators that require validation through longer-duration, multi-cycle experiments before they can be confidently generalized to practical continuous operation. It is also acknowledged that the LCOW values reported for sodium acetate represent an upper bound under RO regeneration and should be reinterpreted if a lower-cost thermal or precipitation route is available for the specific solute chemistry. Site-specific economic evaluation should therefore reassess the regeneration route for sodium acetate before applying these results to a real installation. Performance was further characterized at a single initial osmotic pressure of 30 bar; the concentration-dependent performance envelope of each draw solution across the practical operating range was therefore not mapped, and the comparative rankings reported here should not be extrapolated to other draw solution concentrations without further testing.

Future work should extend the experimental campaign to longer operating duration, validate the *P*_COD_/*K*_foul_ ratio across alternative membrane types and feed matrices, and assess the framework at pilot scale under industrially representative hydraulic conditions.
